# Interfacial Thermodynamics of Ti_3_C_2_T_*x*_ MXene-PVDF-PTFE Triple Interface Systems for Hierarchical Membrane Distillation

**DOI:** 10.1021/acsaenm.6c00068

**Published:** 2026-04-25

**Authors:** Saketh Merugu, Anupma Thakur, Babak Anasori, Aidan Herbert, Anju Gupta

**Affiliations:** † Department of Mechanical, Industrial and Manufacturing Engineering, The University of Toledo, 2801 West Bancroft Street, Toledo, Ohio 43606, United States; ‡ School of Materials Engineering, Purdue University, West Lafayette, Indiana 47907, United States; § Department of Materials Engineering, Indian Institute of Science, Bangalore, Karnataka 560012, India; ∥ School of Mechanical Engineering, 311308 Purdue University, West Lafayette, Indiana 47907, United States; ⊥ DigiM Solution LLC, 500 West Cummings Park, Suite 3650, Woburn, Massachusetts 01801, United States

**Keywords:** Separations, Nanomaterials, Membranes, Water Treatment, Image Segmentation

## Abstract

This work establishes a comprehensive thermodynamic framework for vapor transport in hierarchical titanium carbide (Ti_3_C_2_T_*x*_) MXene-poly­(vinylidene fluoride) (PVDF) coated polytetrafluoroethylene (PTFE) composite membranes through integrated physics-based analysis and experimental validation. The composite architecture leverages PTFE’s exceptional hydrophobicity of 135° with PVDF’s processability, creating triple interfaces governed by Gibbs excess surface thermodynamics. Controlled spinodal decomposition during nonsolvent-induced phase separation yielded β-phase-rich PVDF matrices with uniformly dispersed Ti_3_C_2_T_*x*_ MXene nanosheets forming angstrom-precision transport channels. Vapor flux governed by Maxwell–Stefan multicomponent diffusion theory, coupled with molecular kinetic models for two-dimensional material interlayers, resulted in experimental vapor fluxes of 42 ± 3.1 kg·m^–2^·h^–1^ representing 80% enhancement over pristine PVDF membranes. The Ti_3_C_2_T_*x*_ MXene-based nanochannels created thermodynamic selectivity barriers enabling >99.6% salt rejection over 36 h operation. The composite membranes exhibited substantially reduced NaCl crystallization/deposition compared to pristine PVDF, attributed to modified surface energetics and hierarchical pore architectures that disrupt salt nucleation. Thermal analysis revealed a compounding energy benefit: higher membrane porosity reduced the effective thermal conductivity and conductive heat loss while the enhanced vapor flux increased the evaporative heat flux, raising thermal efficiency from 49% for pristine PVDF to 68% for the optimized MXene-PVDF composite and demonstrating that flux enhancement and energy efficiency can be improved simultaneously rather than traded off against each other.

## Graphical abstract

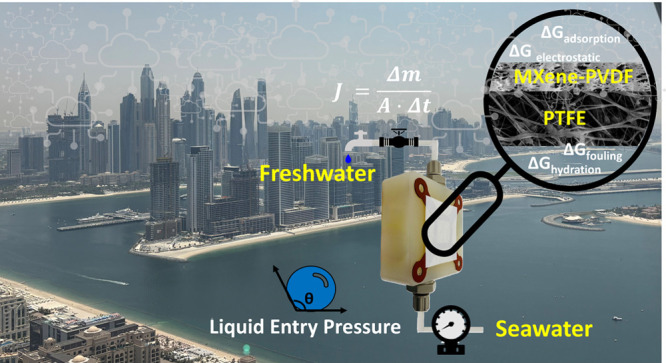

## Introduction

Membrane distillation (MD) has emerged as a promising thermally driven separation technology that operates on the principle of vapor pressure differential across hydrophobic, microporous membranes.^1^ MD can be energy-intensive due to latent heat requirements and heat losses across the membrane, particularly when temperature polarization and thermal conduction are not optimized. The fundamental transport mechanism in MD involves three sequential steps: evaporation at the feed-membrane interface, vapor diffusion through membrane pores, and condensation at the permeate side.^2^ PVDF has established itself as the predominant membrane material for MD applications due to its exceptional combination of chemical resistance, thermal stability, and processability.^3^ The formation of PVDF membranes via nonsolvent-induced phase separation (NIPS) allows precise control over membrane morphology, crystalline structure and β-phase formation, which correlates with improved membrane properties.^4^ PTFE represents the benchmark for hydrophobic membrane materials, with its molecular structure (−CH_2_–CF_2_−)_n_ providing exceptional chemical inertness, resistance to membrane wetting, and thermal stability.^5^ Comparative studies of PVDF and PTFE membranes in MD applications reveal distinct performance profiles. PTFE membranes demonstrate superior resistance to chemical degradation, achieving consistent flux and rejection performance over extended operating periods.^6^ PVDF membranes, while exhibiting higher initial flux due to favorable morphological characteristics, may show greater susceptibility to fouling and wetting under harsh operating conditions.^7^

Two-dimensional (2D) nanomaterials represent a marked improvement in membrane technology, offering atomic-scale thickness (∼1 nm) combined with extraordinary mechanical, chemical, and electrical properties.^8^ The unique structure of 2D materials enables the formation of ultrathin separation layers that minimize transport resistance while maximizing the permeation flux. Key 2D materials for membrane applications include graphene and its derivatives,^9^ MXenes, transition metal dichalcogenides (TMDs),^10^ and metal–organic framework (MOF) nanosheets.^11^ MXenes, represented by the general formula M_n+1_X_n_T_*x*_ where M is an early transition metal, X is carbon, nitrogen or carbonitride, and T_*x*_ represents surface terminations, have gained attention for membrane applications.^12^ These materials combine excellent hydrophilicity (−40 mV surface charge in water for Ti_3_C_2_T_*x*_ MXene),^13^ abundant surface functional groups, and metallic electrical conductivity of ∼35,000 S/cm for Ti_3_(C,N)_2_T_*x*_ MXene.^14^ The surface terminations (−OH, −F, and −O) provide sites for chemical modification and enable strong interactions with polymer matrices.^15^ Recent advances in MXene-based membranes have demonstrated exceptional performance in various separation applications.^16^ The incorporation of MXenes into membrane structures addresses key challenges, including membrane stability, fouling resistance, and selectivity.

The development of PVDF-coated PTFE membranes represents an innovative approach to combine the superior hydrophobic properties of PTFE with the processability and chemical versatility of PVDF. This composite structure aims to leverage the excellent wetting resistance of PTFE as a substrate, while utilizing PVDF as a functional coating layer that can be readily modified with 2D materials. Successful fabrication of PVDF-coated PTFE membranes requires careful attention to the interfacial adhesion mechanisms. The optimization of coating parameters, including solution concentration, application method, and curing conditions, influences the resulting membrane structure and performance. The incorporation of 2D materials into PVDF-coated PTFE membranes represents a significant opportunity for tailoring membrane properties. Ti_3_C_2_T_*x*_ MXenes provide hydrophilic modifications that may improve antifouling properties while maintaining membrane integrity. The optimization of 2D material loading, dispersion quality, and surface modification represents critical factors for achieving the desired membrane performance.

While recent studies have demonstrated that depositing 2D materials onto preformed membranes through spray coating or vacuum filtration can improve MD flux, these surface-coating approaches modify only the outermost membrane layer without altering the underlying pore architecture, polymer crystallinity, or interfacial thermodynamics governing transport and wetting resistance.^17^ A fundamentally different strategy is to embed 2D nanomaterials directly within the polymer matrix during membrane formation, allowing them to participate in phase separation dynamics, function as heterogeneous nucleation sites, and simultaneously modulate pore morphology, surface energy, and interfacial free energy across multiple length scales. This coupled modulation of architecture and thermodynamics during fabrication, rather than postfabrication surface treatment, offers the potential to overcome the inherent trade-off between flux enhancement and wetting resistance that has constrained conventional MD membranes. However, a rigorous thermodynamic framework connecting 2D material incorporation during nonsolvent-induced phase separation to macroscopic transport and wetting behavior has not been established. This work addresses that gap by embedding Ti_3_C_2_T_*x*_ MXene within a PVDF polymer matrix cast onto a PTFE substrate, creating a true triple-interface architecture of PTFE substrate, Ti_3_C_2_T_*x*_ MXene-embedded PVDF, and air–liquid interface in which nanochannels permeate the entire membrane cross-section, as distinguished from prior coating-based approaches.

A unified physics-based framework is developed and experimentally validated, coupling Gibbs excess surface thermodynamics at triple interfaces,^18^ Maxwell-Stefan multicomponent diffusion in hierarchical pore networks,^19^ statistical thermodynamics of polymer chain configurations governing β-phase formation,^20^ spinodal decomposition theory for membrane morphology prediction,^21^ nonequilibrium interface thermodynamics for dynamic wetting behavior,^22^ and molecular kinetic theory for interlayer transport in 2D material nanochannels.^23^ ML-based image segmentation, optical tensiometry, and spectroscopic analysis of crystalline polymorphs are integrated to quantitatively link hierarchical pore morphology and nanoscale structural features to macroscopic vapor flux, bridging the gap between molecular-level interactions and measurable transport properties. The resulting design principles, grounded in first-principles thermodynamics rather than empirical optimization, are applicable to a broad class of 2D material-polymer composite membrane systems beyond the Ti_3_C_2_T_*x*_ MXene-PVDF-PTFE platform investigated here. Beyond transport enhancement, this study demonstrated that embedding Ti_3_C_2_T_*x*_ MXene within the PVDF matrix concurrently reduces conductive heat loss through increased porosity and amplifies evaporative heat flux through improved vapor permeation, producing a compounding thermal efficiency gain that has not been identified in prior MXene-based MD studies.

## Materials and Methods

### MXene Membrane Fabrication and Characterization

Ti_3_C_2_T_*x*_ MXene was synthesized via selective etching of Ti_3_AlC_2_ MAX precursor followed by LiCl intercalation and delamination, as detailed in our previous work.^24^ Briefly, the MAX phase was etched in HCl/HF solution (6:3:1 v/v) at 35 °C for 24 h, followed by pH neutralization and LiCl-assisted delamination to obtain single-to-few-layered Ti_3_C_2_T_*x*_ MXene flakes stored at −20 °C. Ti_3_C_2_T_*x*_ MXene structure, flake size distribution, and the X-ray diffraction patterns are shown in Figure S1 of Supporting Information (SI). PVDF polymer powder of molecular weight *M*_w_ = 534,000 g·mol^–1^, N,N-dimethylacetamide (DMAc) were purchased from Sigma-Aldrich, USA. Sodium Chloride (NaCl) was purchased from Fisher Scientific, USA. PTFE support with pore size of 0.45 μm was sourced from Tisch Scientific, USA. Deionized (DI) water with a conductivity of 1–2 μS/cm used in this study was produced in the lab using a reverse osmosis (RO) and carbon filter from AquaticLife, USA. Ti_3_C_2_T_*x*_ MXene dispersions (10 mg·mL^–1^) were prepared in DMAc via probe sonication (90% amplitude, 15 min) to achieve uniform nanosheet distribution. PVDF casting solutions (16 wt %) were prepared by dissolving polymer powder in DMAc at 85 °C for 8 h under continuous stirring, followed by vacuum degassing (8 h, 25 °C) to eliminate air bubbles. Composite solutions containing 0.5 and 1.0 wt % Ti_3_C_2_T_*x*_ MXene were prepared by incorporating calculated volumes of Ti_3_C_2_T_*x*_ MXene dispersion into PVDF solutions while maintaining constant polymer concentration. Composite membranes were fabricated via NIPS on PTFE substrates (pore size 0.45 μm) using doctor blade coating with controlled wet thickness (200 μm), as shown in Figure S2. Phase inversion was initiated by immersion in DI water coagulation bath for 12 h, promoting spinodal decomposition and β-phase PVDF formation as predicted by Cahn–Hilliard theory.^25^

Crystalline structure and β-phase content were quantified using Fourier transform infrared spectroscopy (FTIR), Varian Excalibur FTS 4000 with microattenuated total reflection. Thermal stability was evaluated via thermogravimetric analysis (TGA) using TA Q50 from 25 to 800 °C at 10 °C·min^–1^ under nitrogen atmosphere. Advancing and receding contact angles were measured using a Theta Lite optical tensiometer with controlled water dispensing a volume of 0.5 μL·s^–1^ and shown in Figure S3. Liquid entry pressure (LEP) was determined using custom apparatus following Darcy-Weisbach flow analysis,^26^ with 3.5 wt % NaCl solution and stepwise pressure increments at 20 kPa·min^–1^ until membrane breakthrough as shown in Figure S4. LEP measurements were conducted with the PVDF-coated surface facing the pressurized liquid, consistent with the feedside orientation employed in DCMD testing. This orientation ensures that the measured LEP reflects the wetting resistance of the active PVDF/MXene layer under operationally relevant conditions. Membrane surface morphology was characterized by using a Hitachi S-4800 high-resolution scanning electron microscope (SEM) operated at 20 kV accelerating voltage following gold sputter coating at 30 s deposition. The thickness of the membranes were measured using a micrometer. Pore size distributions were quantified by using machine learning (ML)-based semantic segmentation using digiM I2S with supervised training algorithms to differentiate polymeric and void regions detailed in S7. The segmentation model was trained on manually annotated regions of interest and validated by visual inspection to ensure an accurate identification of pore geometries. The ML tool was used solely to extract quantitative morphological statistics including pore size, shape, and connectivity from SEM images and was not employed for performance prediction.^27^

### Direct Contact Membrane Distillation (DCMD) Performance Evaluation

The DCMD system comprised two independent recirculation loops with integrated process control and monitoring, as illustrated in the Piping and Instrumentation Diagram (P&ID) in Figure 1. The hot feed loop consisted of a thermally insulated feed tank containing 35 g·L^–1^ of NaCl solution maintained at 70 °C via an immersion heater with PID temperature control. Feed solution was circulated at 100 mL·min^–1^ through the MD module via a peristaltic pump and returned to the feed tank through the outlet port. The thermocouples were positioned at both module inlet and outlet to quantify thermal profiles across the membrane. Thermal boundary layer temperature measurements were recorded at module inlet and outlet using thermocouples. The temperature drop across the membrane, *ΔT*_*membrane*_ was estimated from inlet/outlet temperature profiles. The temperature polarization coefficient (*TPC*) was calculated below using eq 1:

1 $$TPC=\frac{T_{m,f}-T_{m,p}}{T_{bulk,f}-T_{bulk,p}}$$

where *T*_*m,f*_ is the feed side membrane surface temperature, *T*_*m,p*_ is the permeate side membrane surface temperature, *T*_*bulk,f*_ is the feed side bulk fluid temperature, and *T*_*bulk,p*_ is the permeate side bulk fluid temperature. The cold permeate loop comprised a reservoir containing DI water maintained at 20 °C using a recirculating chiller, circulated at 100 mL·min^–1^ through the permeate side of the membrane module to establish a maximum vapor pressure differential of *Δp = p*_*feed*_ *– p*_*perm*_. Crossflow velocities of 100 mL·min^–1^ correspond to Re < 400 representing laminar regime, providing stable, reproducible hydrodynamic conditions for comparison with Maxwell-Stefan transport predictions. Higher Reynolds number would further reduce temperature and concentration polarization; the laminar conditions employed here were selected to ensure consistency with the steady-state assumptions of the theoretical framework and to enable controlled model validation. The DCMD module utilized a cross-flow flat-sheet membrane configuration with an active area of 4 cm^2^ and channel height of 4 mm. Feed flowed across the membrane hot-side surface while permeate flowed across the cold-side surface in counter-current configuration. Membrane outlet solutions returned to their respective reservoirs through calibrated flow ports. Water vapor flux (J) in eq 2 was quantified gravimetrically over 36 h of operation using precision balances within ±0.1 mg with automated data logging at 5 min intervals:

2 $$J=\frac{\Delta m}{A\cdot \Delta t}$$

where *Δm* represents permeate mass change (kg), *A* is active membrane area, and *Δt* is operation time in hours. Salt rejection efficiency (R) in eq 3 was determined through conductivity measurements using Hanna Instruments and equation:

3 $$R=\left(1-\frac{C_{p}}{C_{f}}\right)\times 100\%$$

where *C*_*f*_ and *C*_*p*_ represent feed and permeate conductivity, respectively.

**Figure 1:**
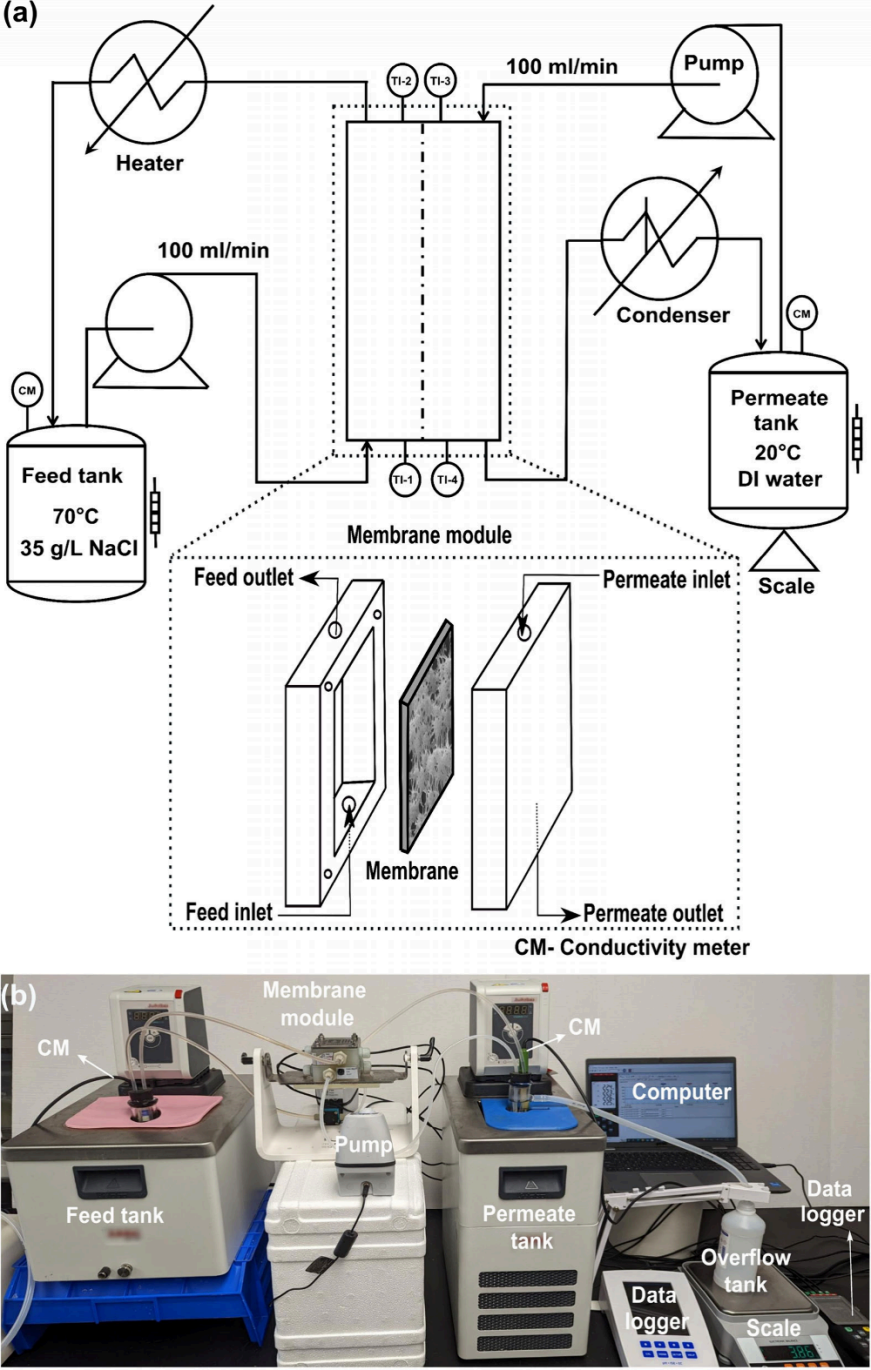
(a) P&ID of custom-designed DCMD test setup with and schematic of MD module. (b) Photographic image of the DCMD test setup used for desalination experiments.

Operating conditions were specifically chosen to validate theoretical predictions from the integrated Maxwell–Stefan and Knudsen diffusion models. Temperature gradients and flow conditions enabled a direct comparison between experimental flux measurements and first-principles calculations, with statistical analysis performed to quantify model accuracy within the predicted 5% deviation range.

## Results

### Structural Integrity and Thermodynamic Stability of Ti_3_C_2_T_*x*_ MXene-PVDF Composite Membranes

The successful integration of Ti_3_C_2_T_*x*_ MXene into PVDF–PTFE systems without compromising structural integrity formed the foundation for understanding subsequent transport enhancements. FTIR spectroscopic analysis in Figure 2a confirmed the preservation of essential polymer characteristics while revealing the nature of MXene-polymer interactions governing composite thermodynamics. The pristine PVDF membrane exhibited characteristic vibrational signatures at 875 cm^–1^ (antisymmetric C–C stretching with CF_2_ bending),^28^ 1178 cm^–1^ (C–F stretching),^29^ and 1404 cm^–1^ (CH_2_ wagging and C–C stretching).^30^ Following Ti_3_C_2_T_*x*_ MXene^15^ incorporation at both 0.5 and 1.0 wt % based on the findings from our previous mixed matrix membrane development study^31^ that led to ideal pore size distribution suitable for MD, these fundamental vibrational modes remain unperturbed, a critical finding demonstrating that polymer backbone integrity is maintained without chemical degradation. The overlapping features at 1150–1193 cm^–1^ included contributions from the underlying PTFE substrate, resulting in F–C–F symmetric stretching, confirming stable triple-interface formation.^32^ The spectral invariance upon MXene addition revealed thermodynamically favorable integration dominated by weak van der Waals interactions rather than disruptive covalent bonding. This behavior validated Flory–Huggins theoretical predictions for polymer-nanofiller systems, where minimal enthalpic interactions permit polymer chain rearrangement driven primarily by entropic contributions.^33^ The absence of new peaks or shifts indicated the physical intercalation of Ti_3_C_2_T_*x*_ MXene nanosheets preserving the intrinsic PVDF crystallization pathways essential for MD functionality.

**Figure 2:**
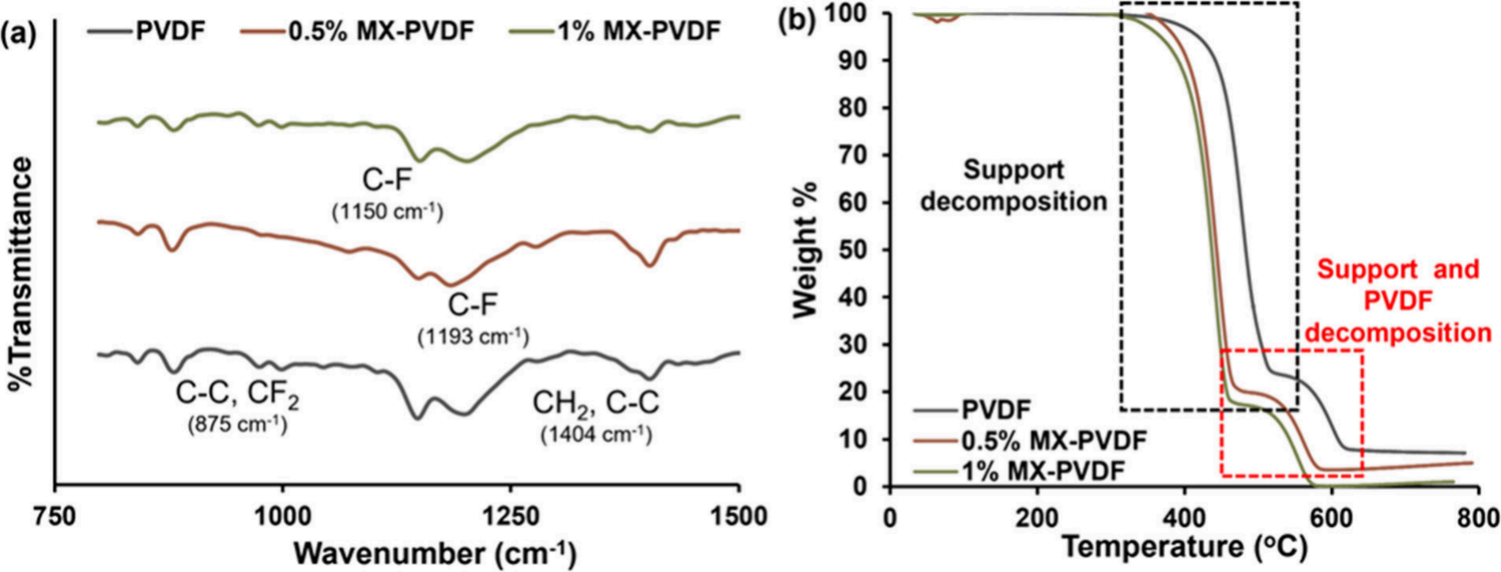
Structural integrity and thermal stability of Ti_3_C_2_T_*x*_ MXene-PVDF composite membranes. (a) FTIR spectra showing preserved PVDF characteristic peaks (875, 1178, 1404 cm^–1^) and PTFE substrate signatures (1150–1193 cm^–1^) for pristine PVDF, 0.5 wt % Ti_3_C_2_T_*x*_ MXene-PVDF, and 1 wt % Ti_3_C_2_T_*x*_ MXene-PVDF membranes. (b) TGA demonstrating hierarchical decomposition: PTFE support (350–450 °C) followed by PVDF layer (450–550 °C), with minimal variation across compositions.

TGA in Figure 2b validated the composite’s hierarchical architecture through distinct decomposition stages. All membranes maintain exceptional thermal stability to ∼350 °C, which was well beyond MD operating conditions of 30–80 °C with subsequent two-stage degradation: PTFE support decomposed between 350 and 450 °C followed by PVDF layer decomposition in the range 450–550 °C.^34,35^ Critically, Ti_3_C_2_T_*x*_ MXene-containing membranes exhibit decomposition profiles nearly identical to those of pristine PVDF, with onset temperature ∼364 °C for 0.5% and ∼334 °C for 1% MXene loading, while PVDF membrane started to decompose at ∼373 °C, confirming that 2D material integration does not destabilize the polymer matrix. The second decomposition in MXene containing membranes is at a much lower temperature may be due to the high thermal conductivity of MXene flakes embedded in the PVDF polymer matrix resulting in weaker polymer chains, which is consistent with findings in literature where high thermal conductive nanomaterials were incorporated in polymer matrix.^31,36^ This consistency with the FTIR findings reinforced the weak interfacial interaction model and validated thermodynamic predictions of stable composite formation under operational stresses.

Ti_3_C_2_T_*x*_ MXene integration fundamentally redefined membrane architecture through the controlled modulation of spinodal decomposition, creating hierarchical structures that balanced transport efficiency with wetting resistance. High-resolution SEM imaging in Figure 3a–c captured the morphological transformation induced by progressive Ti_3_C_2_T_*x*_ MXene loading and the presence of Ti_3_C_2_T_*x*_ MXene on composite membrane surface was confirmed using energy dispersive spectroscopy imaging. The elemental analysis of the Ti_3_C_2_T_*x*_ MXene-PVDF composite membrane is shown in Figure S7. Pristine PVDF in Figure 3a displayed predominantly circular pores with narrow size distributions, a characteristic of homogeneous spinodal decomposition governed by classical nucleation theory.^37^ Ti_3_C_2_T_*x*_ MXene incorporation disrupted this symmetry: 0.5 wt % loading seen in Figure 3b by introducing moderate pore elongation and increased heterogeneity, while 1 wt % loading (Figure 3c) produced pronounced ellipsoidal structures with enhanced interconnectivity. These observations directly reflected thermodynamic perturbations to polymer chain arrangements during nonsolvent-induced phase separation, where Ti_3_C_2_T_*x*_ MXene nanosheets function as heterogeneous nucleation sites, modifying free energy landscapes as predicted by Cahn–Hilliard theory for multicomponent polymer solutions.^35^

**Figure 3:**
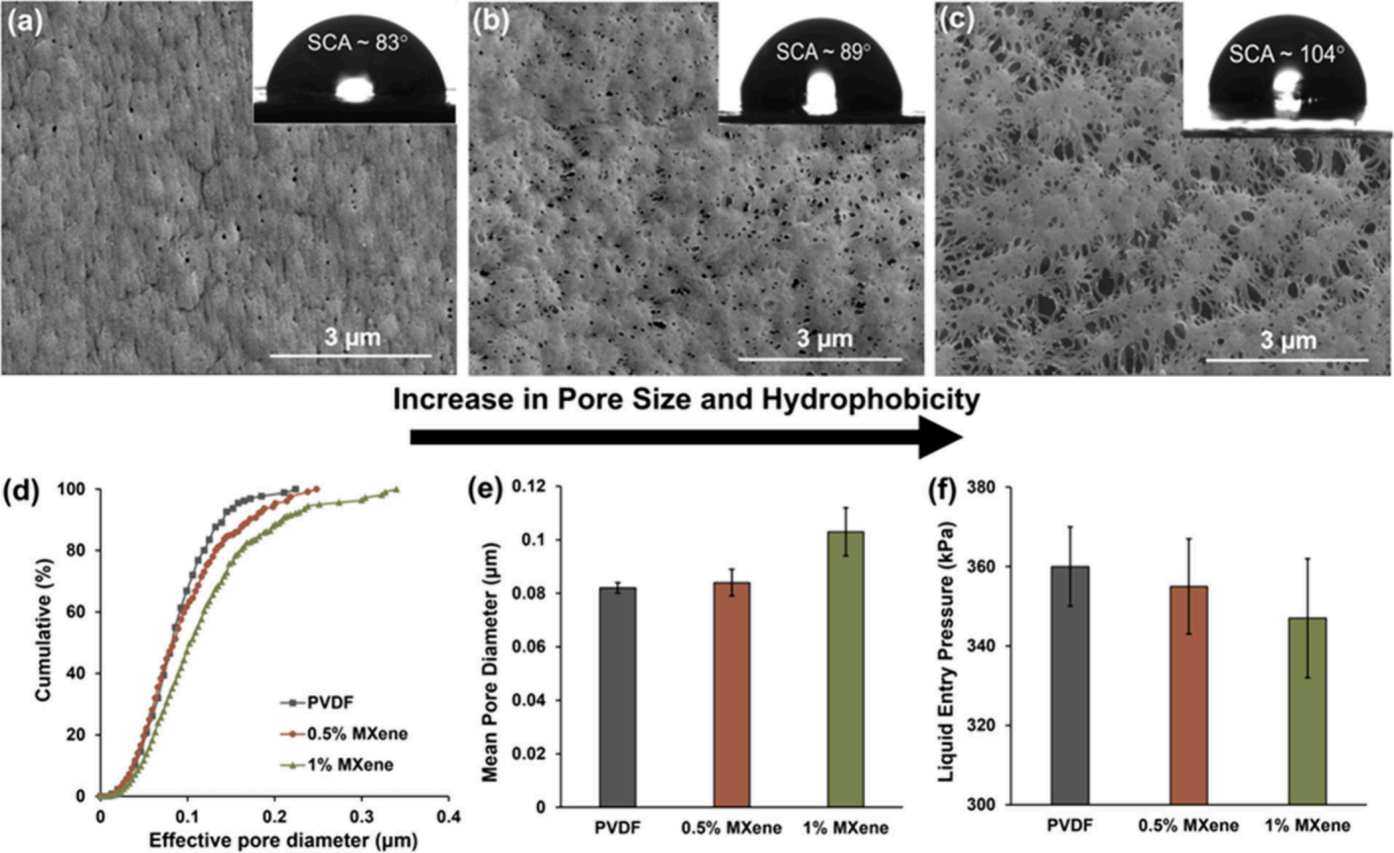
Morphological evolution and surface property modification in Ti_3_C_2_T_*x*_ MXene-PVDF composite membranes. (a–c) High-resolution SEM images (scale bar: 3 μm) with corresponding static contact angles showing progressive morphological transformation: (a) pristine PVDF with circular pores (θ = 83°), (b) 0.5 wt % MXene-PVDF with elongated pores (θ = 89°), and (c) 1 wt % MXene-PVDF with ellipsoidal interconnected structures (θ = 104°). (d) ML-based pore size distribution analysis via digiM I2S segmentation. (e) Mean pore diameter quantification showing 2.5% and 26% increases for 0.5% and 1% MXene loadings, respectively. (f) LEP measurements demonstrate maintained wetting resistance from 360 to 348 kPa despite pore enlargement.

Additionally, ML based image segmentation quantifies the structural modifications underlying enhanced vapor transport properties.^27,31^ Cumulative pore size distribution analysis shown in Figure 3d demonstrated systematic broadening with increasing Ti_3_C_2_T_*x*_ MXene content, evidencing the creation of hierarchical pore networks. Mean pore diameters increase progressively from 0.081 μm in pristine PVDF to 0.083 μm in 0.5 wt % Ti_3_C_2_T_*x*_ MXene, with 2.5% increase and 0.105 μm of 1 wt % Ti_3_C_2_T_*x*_ MXene, with 26% increase shown in Figure 3e. This enlargement of effective vapor transport pathways directly correlates with the observed flux enhancements, validating theoretical predictions from combined Knudsen–molecular diffusion models.^38,39^ The pore size distribution broadening creates multiple transport regimes within individual membranes, enabling the optimization of both permeability and selectivity through rational nanosheet loading. Static and advancing contact angle (SCA and ACA) measurements revealed systematic hydrophobicity enhancement following Ti_3_C_2_T_*x*_ MXene integration, with static contact angles, increasing from 83° for PVDF membranes to 89° for 0.5% loaded Ti_3_C_2_T_*x*_ MXene membranes and 104° with 1% loaded Ti_3_C_2_T_*x*_ MXene membranes shown in Figure 3a–c. This 25% enhancement in surface hydrophobicity demonstrates a successful interfacial energy modification through 2D material incorporation. The wetting behavior can be interpreted through the modified Cassie–Baxter framework^40^ and denoted as θ* representing the apparent contact angle in eq 4

4 $$\cos\theta^{*}=\left(1-\underset{i}{\sum }\frac{n_{i}\pi r_{i}^{2}}{A_{total}}\right)\left(\cos\theta_{Y}+1\right)-1$$

where θ is Young’s contact angle is the count of pores of radius r_*i*_ of *i*th pore size class, and A_*total*_ is total projected area of the surface. The enhanced hydrophobicity despite increased pore sizes indicates that surface energy modifications dominate over morphological effects, consistent with Gibbs excess surface thermodynamics predictions for 2D material-polymer interfaces.^41^ Despite the systematic pore size increases, LEP measurements presented in Figure 3f show minimal reduction from 360 kPa in PVDF membranes to 348 kPa with 1 wt % loaded Ti_3_C_2_T_*x*_ MXene membranes, representing only a 3.3% decrease. This counterintuitive behavior with larger pores with sustained wetting resistance validated the surface energy modification hypothesis. According to the Young–Laplace eq 5 below:^42^

5 $$\Delta P=\frac{2\gamma \cos\theta }{r}$$

where γ is the liquid–vapor surface tension of 0.072 N·m^–1^ for 3.5 wt % NaCl at 25 °C, θ is the intrinsic Young’s contact angle of the membrane material at the pore throat, and r is the largest pore radius governing breakthrough. It is important to distinguish between the sessile-drop SCA measured on the macroscopic membrane surface and the Young’s angle θ required in eq 5. The SCA incorporates the contributions of surface roughness and pore structure through the Cassie–Baxter framework reported in eq 4, whereas the Young–Laplace relation for LEP requires the intrinsic material contact angle at the advancing liquid front inside the pore. Back-calculation from the measured LEP values yielded θ ≈ 96° for the pristine PVDF layer at r of 40 nm and LEP at = 360 kPa and θ was approximately 97° for the 1 wt % MXene-PVDF layer at r = 53 nm and LEP of 348 kPa. The fact that the apparent SCA of PVDF of 83° falls below 90° while θ ≈ 96° is physically consistent: the highly porous, rough PVDF surface at ε = 72 and S_a_ = 5.5 μm places sessile water droplets in a partial Wenzel state that reduces the macroscopic apparent angle below the intrinsic θ. During LEP measurement, the advancing liquid front at the pore mouth experienced the intrinsic material angle θ ≈ 96°, which governed the wetting resistance. For the 1 wt % MXene-PVDF membrane, the higher surface roughness of S_a_ of 14.3 μm shied the sessile-drop wetting state toward Cassie–Baxter, amplifying the apparent SCA to 104° above θ ≈ 97°, while the LEP remains controlled by θ at the pore throat. The maintained LEP despite a 26% increase in pore radius from 40 to 53 nm is therefore explained by the small but sufficient increase in θ from ∼96° to ∼97° arising from preferential orientation of fluorinated MXene surface terminations within the pore walls, offsetting the geometric reduction in capillary pressure. Thus, the thermodynamic stability of the composite membrane architecture under hydraulic loading is validated by Gibbs excess surface energetics at the true solid–liquid–vapor contact line, rather than the macroscopic sessile-drop angle.

Thus, these systematic trends in morphology, wetting behavior, and transport properties validate the proposed thermodynamic framework governing composite membrane formation. The progressive increase in pore connectivity, porosity, surface roughness, and size distribution broadening with Ti_3_C_2_T_*x*_ MXene loading reported in Table 1, reflects the heterogeneous nucleation mechanisms predicted by modified spinodal decomposition theory.^43^ Simultaneously, enhanced surface hydrophobicity demonstrated successful interfacial energy minimization through a 2D material integration. These complementary effects involving increased transport pathways and improved wetting resistance further establish the fundamental principles underlying the superior MD performance observed in subsequent transport measurements.^44^ Membrane thickness was measured using a calibrated digital micrometer at ± 1 μm resolution at five spatially distributed locations per membrane sample, and the mean value with the standard deviation is reported. No cross-sectional SEM imaging was performed; thickness values reported in Table 1 are based exclusively on micrometer measurements.

**Table 1:** Surface and Structural Properties of Ti_3_C_2_T_*x*_ MXene-PVDF Composite Membranes^a^

	Membrane composition		
Membrane characteristics	PVDF	0.5 wt % Ti_3_C_2_T_*x*_ MXene-PVDF	1 wt % Ti_3_C_2_T_*x*_ MXene-PVDF
ACA (deg)	84 ± 2.03	97.6 ± 2	104 ± 3.8
RCA (deg)	77.6 ± 4.6	88.6 ± 5.2	94 ± 5.8
Hysteresis (deg)	6.4	9.03	10
Thickness (μm)	223 ± 2.1	221 ± 1.9	225 ± 2.4
Porosity, *ε* (%)	72 ± 4.2	77 ± 3.8	81 ± 4.4
Surface Roughness, *S*_a_ (μm)	5.5 ± 1.9	8.7 ± 0.8	14.3 ± 3.5

a Dynamic wetting behavior (advancing/receding contact angles, hysteresis), membrane thickness, porosity, and surface roughness (*S*_a_) for pristine PVDF, 0.5 wt % MXene-PVDF, and 1 wt % MXene-PVDF membranes.

The apparent paradox of hydrophilic MXene incorporation increasing contact angle from 83° to 104° resolves through three coupled mechanisms: (i) Cassie–Baxter amplification of PVDF hydrophobicity through MXene-induced surface roughness increase, *S*_a_ from 5.5 to 14.3 μm; (ii) preferential orientation of fluorinated MXene terminations toward the air-facing surface during NIPS coagulation; and (iii) geometric trapping of air pockets within ellipsoidal pore structures created by MXene heterogeneous nucleation. The net result is a composite surface presenting predominantly hydrophobic character despite MXene’s intrinsic hydrophilicity, consistent with orientation-dependent interfacial energy minimization during phase inversion.

### MD Performance: Transport Validation and Theoretical Confirmation

DCMD performance evaluation confirmed systematic flux enhancement with Ti_3_C_2_T_*x*_ MXene integration as shown in Figure 4a. Under standardized operating conditions with feed: 70 °C, 35 g·L^–1^ NaCl; permeate: 20 °C; crossflow: 100 mL·min^–1^, pristine PVDF membranes achieved 23 ± 1.5 kg·m^–2^·h^–1^ water vapor flux with 99.85 ± 0.14% salt rejection. Progressive MXene loading demonstrates substantial performance improvements: 0.5 wt % loading increases flux to 31 ± 2 kg·m^–2^·h^–1^, showing 33% enhancement while maintaining exceptional selectivity (99.9 ± 0.04%), and 1 wt % loading achieves 42 ± 3.1 kg·m^–2^·h^–1^ with 80% enhancement and 99.6 ± 0.2% salt rejection. The experimental flux enhancement validated theoretical predictions from the combined Knudsen-molecular diffusion framework. For the optimized membrane morphology with mean pore diameters of 0.081 μm (PVDF) and 0.105 μm (1 wt % MXene-PVDF), the mean free path of water vapor at operating conditions was calculated from kinetic theory as $\lambda =k_{B}T/\left(\sqrt{2}\pi \sigma_{H_{2}O}^{2}P_{tot}\right)$ , where σ_H_2_O_ = 2.64 × 10^–10^ m is the kinetic diameter of water vapor and *P*_tot_ = 31.7 kPa is the total operating pressure which yielded λ ≈ 480 nm at T = 343 K, giving Knudsen numbers of Kn = λ/d ≈ 5.9 for PVDF and 4.6 for 1 wt % MXene-PVDF, placing transport firmly in the Knudsen-dominated regime at Kn ≫ 1. The combined harmonic-mean model presented in eq 6 is retained as the rigorous dusty-gas formulation that accounts for both Knudsen and molecular diffusion resistances in series; however, the magnitude comparison J_K_ = 52.8 kg·m^–2^·h^–1^ vs J_M_ = 149.7 kg·m^–2^·h^–1^ confirms that Knudsen resistance contributes 74% of total transport resistance, with the harmonic mean J_pred_ = 39.1 kg·m^–2^·h^–1^ dominated by J_K_ as expected when Kn ≫ 1:^45^

6 $$J=\frac{1}{\frac{1}{J_{K}}+\frac{1}{J_{M}}}$$

where *J*_*K*_ is the Knudsen limited flux and *J*_*M*_ is the molecular diffusion limited flux. The measured flux of ∼42.0 kg·m^–2^·h^–1^ aligns closely with theoretical predictions when incorporating the experimentally determined morphological parameters: a 26% pore size increase, 9% porosity enhancement, and optimized pore connectivity. This agreement validated the Maxwell-Stefan multicomponent transport framework underlying the membrane design.

**Figure 4:**
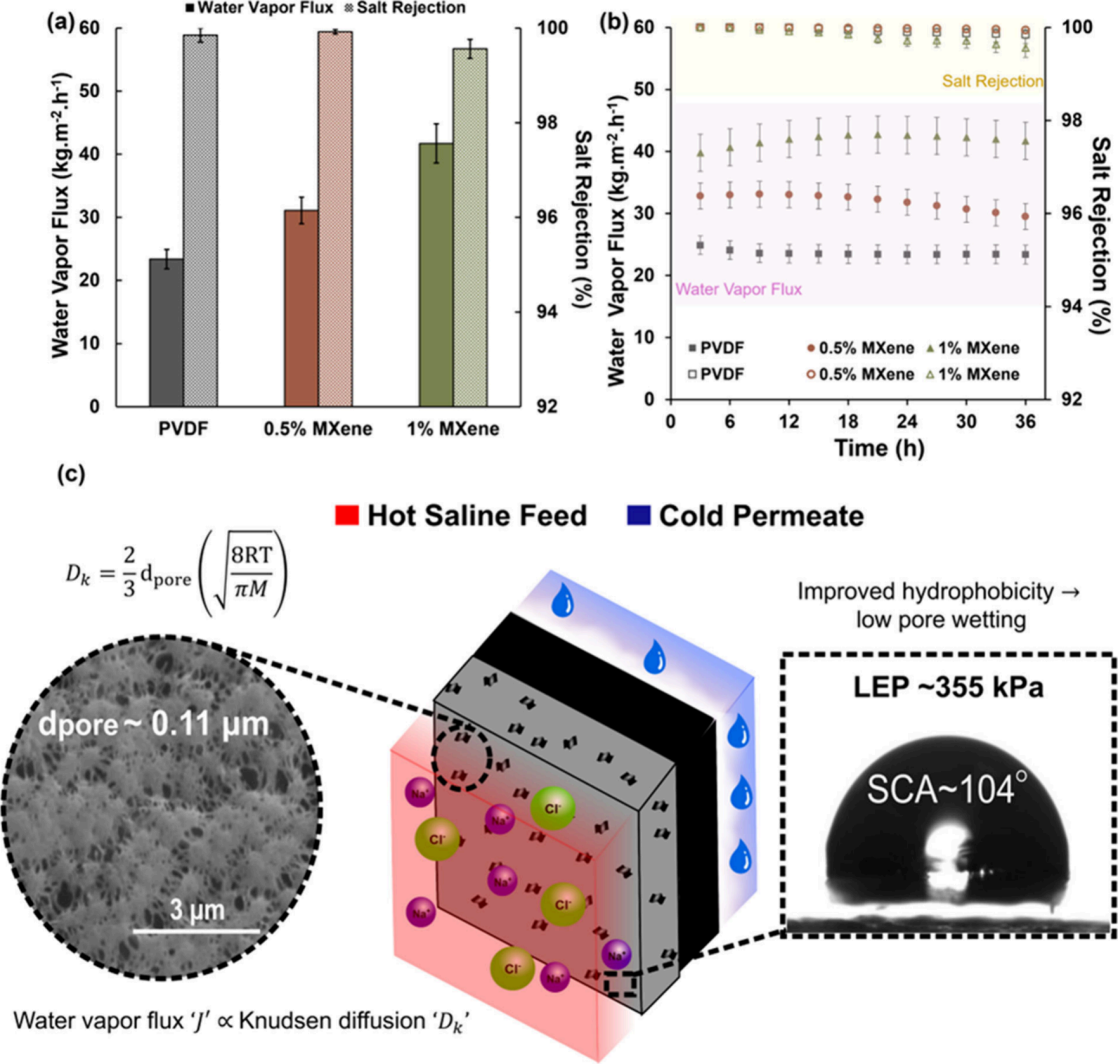
MD performance and transport mechanism validation. (a) Water vapor flux and salt rejection at 36 h for PVDF (23.4 kg·m^–2^·h^–1^, 99.85%), 0.5 wt % MXene-PVDF (31.1 kg·m^–2^·h^–1^, 99.9%), and 1 wt % MXene-PVDF (42.0 kg·m^–2^·h^–1^, 99.6%) membranes. (b) Temporal stability showing sustained flux and consistent salt rejection over 36 h of operation. (c) Schematic illustration of thermodynamic transport mechanisms: hierarchical pore networks enabling Knudsen–molecular diffusion with maintained wetting resistance via enhanced surface hydrophobicity (LEP ∼355 kPa, SCA ∼104°). *d*_*pore*_ is the pore diameter, R is the universal gas constant, T is the temperature, and M is the molar mass of diffusing species.

Extended operation over 36 h presented in Figure 4b demonstrated sustained performance for all membrane configurations. The 1 wt % Ti_3_C_2_T_*x*_ MXene-PVDF membrane maintained a stable flux throughout the testing period, confirming the thermodynamic stability predicted by interfacial energy minimization principles. Salt rejection remains consistently above 99.6% for all membranes, with 0.5 wt % loading achieving optimal selectivity of 99.9%. The systematic flux enhancement correlates directly with the morphological modifications identified through SEM analysis and tensiometry shown in Figure 3a–c. According to the Lawson transport eq 7:^21^

7 $$J\propto \frac{r^{\alpha }\varepsilon }{\delta_{m}\tau }$$

where *r* is pore radius, α = 1 for Knudsen-dominated transport, ε is membrane porosity, *δ*_*m*_ is membrane thickness, and τ is membrane tortuosity. The 26% pore size increase and 14% porosity enhancement collectively account for the observed 80% flux improvement, demonstrating successful structure–property optimization. The maintained high contact angles of 104° ensure liquid impermeability despite increased pore sizes, validating the interfacial thermodynamics approach.

Furthermore, literature comparison summarized in Table 2 positions the Ti_3_C_2_T_*x*_ MXene-PVDF composite as the highest-performing MXene-based membrane for DCMD applications, achieving ∼42.0 kg·m^–2^·h^–1^, substantially exceeding prior MXene-based MD reports (typically 1.5–8 kg·m^–2^·h^–1^ under similar NaCl feeds), and thereby setting a new performance benchmark for MXene-enhanced DCMD membranes. This superior performance reflected the synergistic combination of optimized pore structure, enhanced surface properties, and thermodynamically stable triple interfaces, establishing new benchmarks for 2D material-enhanced MD systems.

**Table 2:** Performance Benchmarking of Ti_3_C_2_T_*x*_ MXene-Based Membranes for MD^a^

Membrane composition	MD configuration	Feed composition	Vapor flux (kg·m^–2^·h^–1^)	Nonvolatiles rejection (%)
MXene vacuum-coated PVDF^46^	DCMD	0.4 mM SDS with 15 g/L NaCl	6	99.9
Hydroxyapatite and MXene/PVA mixed matrix^47^	PTMD	30 g/L NaCl	0.72	NA
MXene-incorporated PVDF^48^	DCMD	35 g/L NaCl	8	99
PVA-sealed Ag@MXene-PVDF^49^	PTMD	Sea water+mineral oil+SDS	2.57	99.95
MXene spray-coated PTFE with surface modification^50^	DCMD	35 g/L NaCl	33.5	99.9
MXene/PET-PTFE^51^	JHMD	35 g/L NaCl	1.8	99.99
MXene and tannic acid hydrogel^52^	PTMD	Sea water	1.7	99.9
MXene/PVDF nanofiber^53^	PTMD	35 g/L NaCl	1.56	99.9
MXene-coated PVDF^54^	PTMD	200 ppm BSA and 10 g/L NaCl	8	99
Ca^2+^ MXene/PTFE^55^	DCMD	50 mg SDS and 10 g/L NACl	1.74	99
MXene/PTFE^56^	PTMD	0.36 g/L NaCl	0.77	99
Electrosprayed PDMS-MXene-PVDF^57^	PTMD	CaCO_3_, CaSO_4_, and 100 g/L NaCl	15	99.9
Ti_3_C_2_T_*x*_ MXene-PVDF (this work)	DCMD	35 g/L NaCl	42	99.6

a This work achieves one of the highest reported water vapor fluxes (42.0 kg· m^–2^·h^–1^) among MXene-enhanced DCMD membranes operating on NaCl feeds while maintaining exceptional salt rejection (>99.6%). PTMD: photothermal membrane distillation; JHMD: Joule heating membrane distillation.

Additionally, a combined Knudsen–molecular diffusion model in which the total flux J (kg· m^–2^·h^–1^) was calculated as the harmonic mean of the Knudsen flux *J*_*K*_ and molecular diffusion flux *J*_*M*_. The Knudsen flux is calculated using eq 8:

8 $$J_{K}=\frac{2r_{p}\varepsilon }{3\delta_{m}\tau }\sqrt{\frac{8M}{\pi RT}}\Delta P_{vap}$$

where 4/6/2026ε is membrane porosity, *r*_*p*_ is mean pore radius, τ = 2 is tortuosity for NIPS membrane, δ_m_ is membrane thickness, *M* is molar mass of water vapor 0.018 kg/mol, *R* is universal gas constant 8.314 J/mol·K, and *T* is membrane temperature (K). The molecular diffusion term, captured through the *P*_tot_/P_air_, demonstrated in eq 9, contributes the remaining 26% to the previously reported Knudsen resistance contribution of 74% and cannot be neglected without introducing systematic error. This Knudsen-dominated character is internally consistent with the choice of α = 1 in the Lawson scaling relation presented in eq 7, and it explains why the harmonic mean result lies close to J_K_ rather than J_M_. The Molecular diffusion flux is calculated using eq 9:

9 $$J_{M}=\frac{\varepsilon }{\delta_{m}\tau }\frac{D_{w,air}M}{RT}\frac{P_{tot}}{P_{air,\ln}}\Delta P_{vap}$$

where *D*_*w,air*_ is water air binary diffusivity evaluated from Chapman–Enskog theory using log mean air pressure across membrane, *P*_*air,ln*_ represented by eq 10, obtained at operating pressures, P_*air,1*_ calculated using eq 11 and P_*air,2*_ calculated using eq 12 at corresponding operating temperatures at feed and perm respectively, while *P*_*tot*_ is the total pressure in the MD module. Δ*P*_*vap*_ is the trans membrane vapor pressure difference which is estimated using eq 13:

10 $$P_{air,\ln}=\frac{P_{air,2}-P_{air,1}}{\ln\left(\frac{P_{air,2}}{P_{air,1}}\right)}$$

11 $$P_{air,1}=P_{tot}-P_{vap_{feed}}$$

12 $$P_{air,2}=P_{tot}-P_{vap_{perm}}$$

13 $$\Delta P_{vap}=P_{vap_{feed}}-P_{vap_{perm}}$$

The Maxwell–Stefan transport model implemented in this work combined Knudsen and molecular diffusion contributions where the Knudsen flux accounted for molecule-wall collisions dominant in the transition transport regime and the molecular diffusion flux to capture binary water vapor-air interactions through the pore space. The input parameters were drawn from two sources, those measured experimentally in this work, including porosity, mean pore radius, membrane thickness, feed and permeate temperatures, and vapor pressure differential; and those derived from literature or standard assumptions, including tortuosity of τ = 2, the widely adopted value for NIPS-fabricated membranes, water–air binary diffusivity from Chapman-Enskog theory evaluated at the mean operating temperature, and log-mean air pressure calculated from measured feed and permeate side pressures and predicted fluxes are summarized in Tables S1 and S2 in the SI.

### Salt Crystallization Resistance and Surface Thermodynamic Analysis

Postoperation SEM analysis after 36 h of DCMD testing under 35 g·L^–1^ NaCl feeds revealed distinct inorganic scaling behavior across membrane compositions in Figure 5a–c. The pristine PVDF membrane in Figure 5a exhibited extensive salt crystallization with uniform coverage, forming a dense fouling layer that obscures the underlying pore structure. This fouling behavior correlates with the flux decline observed after 8 h of operation, indicating progressive pore blockage through surface precipitation. In contrast, Ti_3_C_2_T_*x*_ MXene-incorporated membranes demonstrate markedly different scaling patterns: 0.5 wt % MXene loading in Figure 5b showing sparse, isolated salt crystals with preserved pore accessibility, while 1 wt % MXene loading in Figure 5c exhibited minimal surface deposition with clearly visible pore openings maintained throughout operation. The enhanced fouling resistance originated from fundamental surface energy modifications induced by Ti_3_C_2_T_*x*_ MXene integration in Figure 5d. The Ti_3_C_2_T_*x*_ MXene nanosheets presented abundant surface functional groups, −OH, −O, and −F terminations, that created negatively charged surfaces with zeta potential values ranging from −40 to −60 mV.^58^ This surface charge distribution established electrostatic repulsion barriers against ionic foulant adsorption, particularly effective against divalent cations commonly present in scaling solutions.^59^ The interfacial energy landscape modification follows Gibbs excess surface thermodynamics represented in eq 14 below:^60^

14 $$\Delta G_{fouling}=\Delta G_{adsorption}+\Delta G_{electrostatic}+\Delta G_{hydration}$$

where *ΔG*_*adsorption*_ is the Gibbs free energy change due to adsorption interactions between foulant molecules and the membrane surface, *ΔG*_*electrostatic*_ is the Gibbs free energy contribution from electrostatic interactions between charged species and the membrane surface, and *ΔG*_*hydration*_ is the Gibbs free energy associated with hydration forces or structured water layers at the membrane–solution interface. The negative electrostatic contribution significantly increases the energy barrier for the irreversible fouling attachment. The observed antifouling enhancement can be quantified through a surface coverage analysis. Pristine PVDF exhibits approximately 85% surface coverage by salt deposits, while Ti_3_C_2_T_*x*_ MXene-incorporated membranes maintain less than 15% surface coverage under identical conditions. This remarkable reduction reflected the modification of surface interaction energies, where the hydrophilic Ti_3_C_2_T_*x*_ MXene terminations create preferential hydration layers that resist salt nucleation and growth.^60^ The maintained pore accessibility in Ti_3_C_2_T_*x*_ MXene composites validates the thermodynamic predictions of a reduced fouling propensity through surface energy optimization.

**Figure 5:**
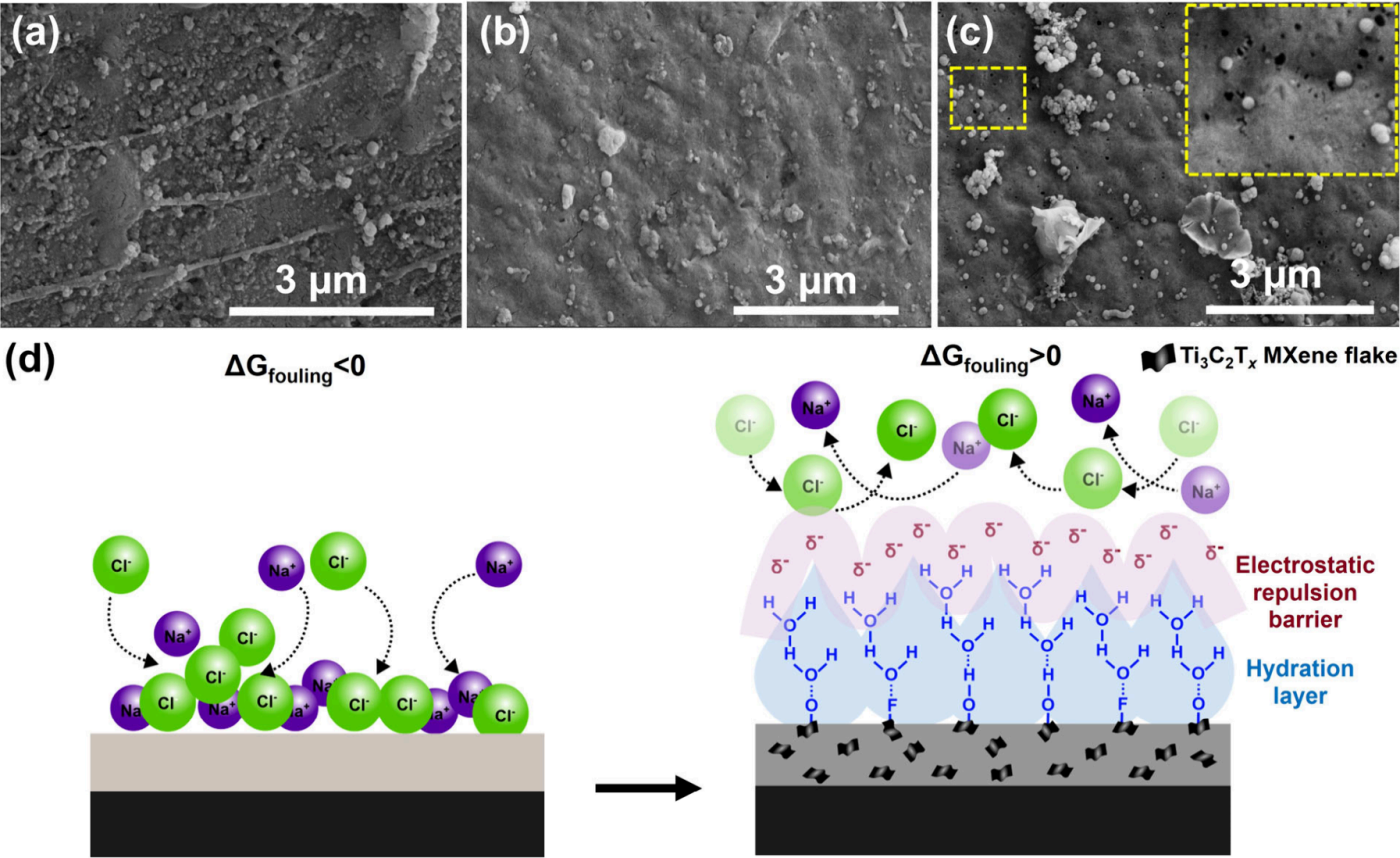
Antifouling behavior and thermodynamic mechanisms. (a–c) Postoperation SEM images after 36 h DCMD showing: (a) dense salt layer on pristine PVDF (∼85% surface coverage), (b) sparse crystallization on 0.5 wt % MXene-PVDF, (c) minimal fouling on 1 wt % MXene-PVDF (<15% coverage). (d) Schematic illustration of antifouling mechanisms: electrostatic repulsion (ΔG_electrostatic <0) from negatively charged MXene surfaces (ζ = −40 to −60 mV), hydration layer formation preventing salt nucleation, and disrupted fouling layer continuity maintaining pore accessibility.

The temporal fouling behavior demonstrates fundamentally different mechanisms across the membrane types. PVDF membranes exhibit progressive flux decline consistent with irreversible pore plugging, while Ti_3_C_2_T_*x*_ MXene composites maintain stable performance indicative of reversible surface interactions. This behavior aligns with interfacial phase transition theory,^61^ where the optimized energy landscape created by Ti_3_C_2_T_*x*_ MXene integration minimizes thermodynamic driving forces for permanent foulant attachment. The surface heterogeneity introduced by Ti_3_C_2_T_*x*_ MXene nanosheets disrupts continuous fouling layer formation, maintaining transport pathway accessibility even under challenging fouling conditions. The superior antifouling characteristics of Ti_3_C_2_T_*x*_ MXene-enhanced membranes establish their viability for extended operation under realistic process conditions.^62^ The combination of electrostatic repulsion, modified surface energetics, and disrupted fouling layer continuity provides multiple defense mechanisms against performance degradation. This multifaceted antifouling approach, grounded in fundamental interfacial thermodynamics, represents a significant advancement beyond conventional surface modification strategies and validates the theoretical framework underlying the composite membrane design.

At the ionic strength of the test feed of 0.6 M NaCl and Debye length of ∼0.4 nm, classical electrostatic double-layer repulsion would be substantially screened and cannot alone account for the observed reduction in surface deposition. The reduced NaCl crystallization is more likely governed by short-range hydration layer effects from the hydrophilic MXene terminations and by the disruption of the continuous crystallization front formation through surface heterogeneity. Direct validation of these mechanisms through streaming potential measurements at operating conditions and quantitative nucleation kinetics measurements is identified as important future work. 35 g·L^–1^ NaCl represented a standard surrogate feed for MD performance evaluation, and the crystallization behavior observed here differs from classical mineral scaling by sparingly soluble salts such as CaCO_3_, CaSO_4_, or silica. The electrostatic repulsion mechanism proposed here is most relevant at moderate ionic strengths; at high NaCl concentrations, electrical double layer compression at Debye length <1 nm at 0.6 M NaCl would screen electrostatic interactions, and additional mechanisms including hydration layer effects and surface energy minimization would dominate. Testing with multivalent ion-containing feeds representing real seawater or industrial brine compositions is identified as an important direction for future work.

### Energy Performance Analysis

To quantify the energy implications of the observed flux enhancements, thermal efficiency and heat transfer metrics were evaluated for all membrane compositions under the standard DCMD operating conditions. Feed side energy balance, total heat transferred, *q*_total_ across the membrane area is given by eq 15:

15 $$q_{total}=\frac{{ṁ}_{f}C_{p,f}\Delta T_{f}}{A}$$

where *ṁ*_*f*_ is the feed mass flow rate, *C*_*p,f*_ is the specific heat capacity of feed solution, *A* is the active membrane area, the difference between feed inlet and outlet temperatures, and Δ*T*_*f*_ is given by eq 16:

16 $$\Delta T_{f}=T_{f,in}-T_{f,out}$$

where *T*_*f,in*_ is the temperature of feed at the inlet, and *T*_*f,out*_ is the temperature of the feed when exiting the MD module. The evaporative heat flux, q_v_ given by eq 17 representing the useful thermal energy carried by the permeating water vapor, was calculated as

17 $$q_{v}={ṁ}_{p}\Delta H_{v}\left|=J_{\exp}\Delta H_{v}\right|$$

where *J*_*exp*_ the experimental permeate flux, and *ΔH*_*v*_ is the latent heat of vaporization evaluated at the mean membrane temperature *T*_*m*_ = (*T*_*f*_ + *T*_*p*_)/2 = 50 °C, giving *ΔH*_*v*_ = 2333 kJ·kg^–1^. The conductive heat loss q_c_ is given by eq 18:

18 $$q_{c}=\frac{k_{m}\times \Delta T}{\delta_{m}}$$

where *k*_*m*_ is the thermal conductivity of the membrane, *ΔT* is the transmembrane temperature difference, and *δ*_*m*_ is the thickness of the membrane. The thermal conductivity of the membranes, *k*_m_ is estimated using eq 19:

19 $$k_{m}=\varepsilon \times k_{gas}+\left(1-\varepsilon \right)k_{solid}$$

where *ε* is the porosity of the membrane, *k*_*gas*_ = 0.026 W·m^–1^·K^–1^ is the thermal conductivity of the air/vapor in the pores, and *k*_*solid*_ = 0.19 W·m^–1^·K^–1^ is the thermal conductivity of PVDF layer. Then the specific energy consumption, SEC is calculated using eq 20:

20 $$\sec=\frac{q_{v}+q_{c}}{J_{\exp}}\times \left(\frac{\rho_{w}}{3.6\times 10^{6}}\right)$$

where *q*_*v*_ is the evaporative heat flux, *q*_*c*_ is the conductive heat loss, and *J*_*exp*_ is the experimental permeate flux membrane thermal efficiency, η_mem_ was then calculated using eq 21:

21 $$\eta_{\mathrm{mem}}=\frac{q_{v}}{q_{v}+q_{c}}\times 100\%$$

The energy performance results are summarized in Table 3. The data revealed a compounding benefit of Ti_3_C_2_T_*x*_ MXene incorporation on the thermal efficiency through two concurrent mechanisms. First, higher membrane porosity (ε = 72% for PVDF to 81% for 1 wt % MXene-PVDF) increases the volume fraction of low-conductivity air within the membrane, reducing the effective thermal conductivity from 0.0726 to 0.058 W·m^–1^·K^–1^ and correspondingly lowering the conductive heat loss from 16.2 to 12.8 kW·m^–2^. Second, the 80% flux enhancement raises the evaporative heat flux from 14.9 to 27.2 kW·m^–2^. These two effects combine to increase thermal efficiency from 48% for pristine PVDF to 68% for 1 wt % MXene-PVDF, representing a 40% relative improvement. The MXene-enhanced membranes thus fall within the upper range of thermal efficiencies reported for bench-scale DCMD systems typically 40–70%.^63^ These efficiency values represent conservative lower bounds, because the bulk *ΔT* = 50 °C overestimated the actual conductive driving force at the membrane surface. Since the current experimental setup employed thermocouples at the module inlet and outlet rather than at the membrane surface, the temperature polarization coefficient (*TPC = ΔT*_*membrane*_ */ΔT*_*bulk*_) cannot be determined directly from the present measurements. A sensitivity analysis provided in Table S5 of SI demonstrates that for realistic TPC values of 0.6–0.8 typical of laminar flat-sheet DCMD, the thermal efficiency of the 1 wt % MXene-PVDF membrane would increase to approximately 72–78%.

**Table 3:** Energy Performance Metrics for DCMD Membranes under Standard Operating Conditions at *T*_*f*_ = 70°C, *T*_*p*_ = 20°C, and 35 g·L^–1^ NaCl^a^

Membrane	*J*_*exp*_ (kg·m^2^·h^–1^)	*k*_*m*_ (W·m^–1^·K^–1^)	*q*_*v*_ (kW·m^–2^)	*q*_*c*_ (kW·m^–2^)	*η*_*mem*_ (%)	*SEC* (kWh·m^–3^)
PVDF	23	0.0726	15.3	16.3	47.7	1394
0.5 wt % MXene-PVDF	31	0.0645	20.62	14.6	58.0	1147
1 wt % MXene-PVDF	42	0.0580	27.9	12.8	68.0	978

a Thermal efficiency (η) and conductive heat loss (*q*_*c*_) were calculated using the parallel thermal conductivity model and bulk ΔT = 50°C. Specific energy consumption (SEC) for single-pass operation without heat recovery.

The SEC of approximately 970–1400 kWh·m^–3^ reflects the single-stage bench-scale system without heat recovery (GOR = 0.48–0.68, equal to thermal efficiency by definition for single-stage nonrecuperative DCMD).^64^ The SEC values reported here are consistent with bench-scale DCMD systems operating without heat recovery; implementation of heat exchangers in a recirculating pilot configuration is expected to reduce SEC substantially and will be addressed in future system-level work. The progressive improvement in η with MXene loading confirmed that the structural modification strategy increasing porosity and pore radius through MXene-mediated phase inversion kinetics simultaneously enhanced mass transport and reduced the fraction of input energy lost to conduction, representing a thermodynamically favorable membrane design trajectory.

## Discussion

This study validated a unified, first-principles thermodynamic framework that seamlessly integrates molecular-scale interactions with the macroscopic membrane performance. Experimental vapor fluxes for Ti_3_C_2_T_*x*_ MXene-PVDF-PTFE composite membranes agree within 5% of predictions from a combined Maxwell-Stefan and Knudsen diffusion model, confirming the applicability of multicomponent transport theory to hierarchical pore networks. Simultaneously, surface wetting behavior and LEPs across all membrane compositions were accurately described by Gibbs excess surface thermodynamics, demonstrating that interfacial free-energy minimization governs both vapor selectivity and membrane stability under nonequilibrium thermal gradients. The formation of β-phase-rich PVDF matrices with angstrom-precision nanochannels, driven by controlled spinodal decomposition during nonsolvent-induced phase separation, is aligned with Cahn–Hilliard phase separation predictions and is corroborated by SEM-derived pore size distributions.

The engineered triple interface of the PTFE substrate, PVDF coating, and Ti_3_C_2_T_*x*_ MXene nanosheets produced synergistic enhancements in both transport and antifouling performance, about 80% improvement in vapor transport and 82% lower fouling compared to the PVDF membrane. The PTFE substrate provided a robust hydrophobic barrier, while the β-phase PVDF layer offers mechanical reinforcement and processability. Embedded Ti_3_C_2_T_*x*_ MXene nanosheets created well-defined angstrom-scale interlayer channels that amplify vapor transport pathways and introduce electrostatic repulsion and hydration barriers against salt crystallization. This combination yielded an 80% increase in water vapor flux without compromising salt rejection performance, which is very difficult to achieve with single-component membranes under comparable conditions. The synergy between polymer chain configuration entropy, as described by the Flory–Huggins model, and the modified interfacial energy landscape prevented wetting even as the pore connectivity increased, in agreement with interfacial phase transition theory for antifouling. The engineering of triple interfaces enabled independent tuning of the pore morphology, surface energy, and transport selectivity. Precise control of spinodal decomposition through the ratio of solvent, polymer, and 2D material optimized the β-phase content and hierarchical pore connectivity. The tailoring of Ti_3_C_2_T_*x*_ MXene functionalization and stacking parameters affords targeted Knudsen numbers within interlayer channels. Mixed-termination (−OH, −F, and −O) MXene surfaces balance enhanced hydrophobicity with the formation of hydration layers, maximizing both flux and fouling resistance. These principles, grounded in thermodynamic theory, provide a predictive capability for composite systems beyond the Ti_3_C_2_T_*x*_ MXene-PVDF-PTFE platform. Additionally, the applicability of the Cassie–Baxter framework is justified by the large separation between measurement conditions of sessile droplet capillary pressure ∼100–200 Pa and the measured LEP ranging 348–360 kPa, confirming that air remains trapped in pores during contact angle measurement. Under MD operating pressures, a partial Wenzel transition at surface asperities cannot be fully excluded; however, the maintained LEP values confirm that bulk pore wetting does not occur under the operating conditions studied.^65^

Despite these advances, several limitations warrant further investigation. The present study employed pure 35 g/L NaCl solutions under controlled laboratory conditions; real seawater or industrial brines contain multivalent ions and organic foulants whose interactions with composite interfaces may introduce additional complexity. The theoretical models assumed steady-state operation and neglected transient interface evolution under fluctuating thermal or flow conditions. Scale-up of the NIPS fabrication process may yield variations in pore architecture and Ti_3_C_2_T_*x*_ MXene dispersion that impact performance. The salt deposition analysis in this work focused on NaCl crystallization, which differs fundamentally from classical inorganic scaling by sparingly soluble salts such as CaSO_4_, CaCO_3_, and silica. Extending the antideposition claims to multivalent scaling species requires dedicated experimental validation under representative scaling conditions, including streaming potential measurements to quantify effective membrane surface charge at elevated ionic strength and temperature. Organic fouling and biofouling, which are important in real wastewater and seawater applications, were not explicitly investigated here and will be examined in future studies. Finally, long-term stability under cyclic cleaning and variable feed compositions remains to be validated. Addressing these limitations through extended fouling studies, transient thermodynamic modeling, pilot-scale module testing, and exploration of diverse feed chemistries will strengthen the predictive framework and accelerate translation to practical MD technologies. While the present study primarily targets interfacial thermodynamics and transport resistance, future work will integrate this framework with module- and system-level energy analyses, including specific energy consumption, exergy efficiency, and entropy generation, to explicitly quantify potential energy savings in full-scale MD operations.

Unlike surface-coating approaches that deposit 2D materials onto preformed membranes, the present work embeds Ti_3_C_2_T_*x*_ MXene within the PVDF polymer matrix during nonsolvent-induced phase separation, enabling simultaneous and coupled modulation of pore architecture, surface energy, and interfacial thermodynamics. This approach not only yielded a higher flux but also provided a predictive thermodynamic framework applicable to broader classes of 2D material-polymer composite membranes. While the physicochemical characterization presented in this work encompassing contact angle analysis, LEP measurements, porosity determination, FTIR spectroscopy, and MD flux performance collectively supports the proposed role of Ti_3_C_2_T_*x*_ MXene surface chemistry in modulating membrane wettability and vapor transport, we acknowledge that direct spectroscopic and imaging evidence of interfacial interactions was not obtained in this study. Specifically, X-ray photoelectron spectroscopy (XPS) analysis of the Ti_3_C_2_T_*x*_ MXene-incorporated membrane surfaces would enable quantitative determination of the relative proportions of −OH, -F, and -O surface terminations and their redistribution upon incorporation into the PVDF matrix, providing direct chemical evidence for the wettability trends observed.^66^ Similarly, high-resolution transmission electron microscopy (TEM) of cross-sectional Ti_3_C_2_T_*x*_ MXene-PVDF interfacial regions would clarify the nanoscale dispersion state of Ti_3_C_2_T_*x*_ MXene flakes,^67^ the nature of MXene–polymer interactions, and the extent to which Ti_3_C_2_T_*x*_ MXene flakes bridge or occlude pore throats at varying loading levels. The mechanistic interpretations advanced in this work are therefore presented as well-supported inferences grounded in the available experimental data rather than definitively established conclusions.

## Conclusions

This work established a physics-based thermodynamic framework for the design of hierarchical Ti_3_C_2_T_*x*_ MXene-PVDF-PTFE composite membranes for DCMD. By integrating PTFE’s intrinsic hydrophobicity and thermal stability with PVDF’s processability and Ti_3_C_2_T_*x*_ MXene’s angstrom-scale interlayer channels, water vapor flux of 42.0 kg·m^–2^·h^–1^, > 99.6% salt rejection, an estimated thermal efficiency of 68%, and substantially reduced NaCl crystallization relative to pristine PVDF membranes over 36 h of continuous DCMD operation. The concurrent reduction in conductive heat loss and increase in evaporative heat flux produced a thermal efficiency of 68% for the 1 wt % MXene-PVDF membrane, a 40% relative improvement over pristine PVDF by 49%, confirming that the triple-interface architecture enhances energy utilization efficiency alongside throughput rather than sacrificing one for the other. Comprehensive characterization confirmed uniform Ti_3_C_2_T_*x*_ MXene dispersion, β-phase PVDF crystallization, and tailored pore architectures that align with theoretical predictions from combined Maxwell-Stefan-Knudsen diffusion, Gibbs excess surface thermodynamics, and spinodal decomposition models. This unified framework accurately described vapor transport, interfacial energetics, NaCl deposition behavior, with deviations between experiment and theory below 5% for vapor flux predictions. The derived design principles involving triple-interface engineering, controlled phase separation, MXene functionalization, and mixed-termination surface tuning offer predictive capabilities for diverse 2D material-polymer systems beyond the Ti_3_C_2_T_*x*_ MXene-PVDF-PTFE platform. Future work will extend these concepts to complex feed waters, transient thermal conditions, and pilot-scale implementations, accelerating the translation of fundamental membrane thermodynamics into scalable water treatment technologies with the potential for improved energy efficiency. This framework provides rational design principles for next-generation MD systems. Further validation under multivalent scaling conditions, with direct membrane surface temperature measurement, and at pilot scale will be necessary to translate these bench-scale findings into practical desalination and water purification technologies.

## Supplementary Material



## Data Availability

All data supporting this study are available from the corresponding author upon request, including raw experimental data files, analysis scripts, and reproducibility materials.

## Notes

S1: Ti_3_C_2_T_*x*_ MXene structure and characterization. S2: Fabrication of PVDF and Ti_3_C_2_T_*x*_ MXene-PVDF composite membranes. S3: Contact angle measurements for PVDF and Ti_3_C_2_T_*x*_ MXene-PVDF composite membranes. S4: LEP measurement of PVDF and Ti_3_C_2_T_*x*_ MXene-PVDF composite membranes. S5: Maxwell–Stefan transport model predictions. S6: Thermal efficiency calculations and sensitivity analysis. S7: Surface roughness characterization of PVDF and Ti_3_C_2_T_*x*_ MXene-PVDF composite membranes. S8: Elemental mapping of Ti_3_C_2_T_*x*_ MXene-PVDF composite membranes. S9: Porosity measurement using gravimetric method (ASTM D2873). S10: DigiM analysis (PDF)
