# Supplementary material for: Interfacial Thermodynamics of Ti3C2T x MXene-PVDF-PTFE Triple Interface Systems for Hierarchical Membrane Distillation
Source: ACS Appl Eng Mater. 2026 Apr 25;4(5):2160–73. doi: 10.1021/acsaenm.6c00068 (PMC13200180; doi:10.1021/acsaenm.6c00068)
Supplement: Supplementary file 1 [file em6c00068_si_001.pdf]

Supporting Information

Interfacial Thermodynamics of $Ti_3C_2T_x$ MXene-PVDF-PTFE Triple Interface Systems for Hierarchical Membrane Distillation

*Saketh Merugu[1], Anupma Thakur[2,3], Babak Anasori[2,4], Aidan Herbert[5], Anju Gupta[1] **

[1] Department of Mechanical, Industrial and Manufacturing Engineering, The University of Toledo, 2801 West Bancroft Street, Toledo, OH 43606, United States

[2] School of Materials Engineering, Purdue University, West Lafayette, IN 47907, United States

[3]Department of Materials Engineering, Indian Institute of Science, Bangalore, Karnataka 560012, India

[4]School of Mechanical Engineering, Purdue University, West Lafayette, IN 47907, United States

[5]DigiM Solution LLC, 500 West Cummings Park, Suite 3650, Woburn, MA 01801, United States

*Corresponding author:

Dr. Anju Gupta

E-mail: anju.gupta@utoledo.edu

Department of Mechanical, Industrial and Manufacturing Engineering, University of Toledo, 1610 N Westwood Ave, NI 4055, Toledo, OH 43606.

Telephone: +1 (413) 530-8213

S1. $Ti_3C_2T_x$ MXene Structure and Characterization

Figure S1a illustrates the atomic structure of the $Ti_3C_2T_x$ MXene used in this work, showing its characteristic 2D carbide lattice with mixed surface terminations -OH, -O, and -F[1]. The MXene was synthesized via selective etching of Ti_3AlC_2 MAX phase using LiF/HCl treatment, followed by delamination in dimethyl sulfoxide (DMSO) to yield single-layer nanosheets[2]. To synthesize $Ti_3C_2T_x$ MXene, 1 g of Ti_3AlC_2 MAX was first washed using 9 M HCl obtained from Fisher Scientific for 18 h to remove intermetallic impurities and mixed with an etchant solution at 6:3:1 mixture (by volume) of 12 M HCl, DI water, and 50 wt % HF stirring at 400 RPM for 24 h at 35 °C. The etched $Ti_3C_2T_x$ MXene was washed with deionized water via repeated centrifugation at 3234 RCF at 4-5 cycles with ~200 mL of deionized water until the supernatant reached pH ~6. For delamination, the etched multilayered $Ti_3C_2T_x$ MXene sediment was then added to LiCl typically 50 mL/per gram of starting etched powder solution. The mixture of LiCl and multilayer MXene was then stirred at 400 RPM for 1 h at 65 °C under constant argon gas flow. The mixture was then washed with deionized water via centrifugation at 3234 RCF for 5, 10, 15, and 20 minutes. Then, the final mixture was vortexed for 30 minutes, followed by centrifugation at 2380 RCF for 30 minutes to ensure the $Ti_3C_2T_x$ MXene solutions were single-to-few-layered flakes[2]. Particle size distribution analysis by laser diffraction in Fig S1b revealed a narrow size distribution centered at 5-10 μm with minimal submicron debris, confirming effective delamination while maintaining structural integrity. This well-defined morphology ensured consistent dispersion and integration into PVDF polymer matrices during membrane fabrication. Phase purity and structural integrity were confirmed using Bruker D8 diffractometer powder X-ray diffraction with Cu Kα radiation, λ = 1.5406 Å and a VANTEC 500 detector. Samples were scanned from 5° to 85° 2θ with 0.02° step size and 1s per step integration time, following established protocols[2]. The resulting

XRD patterns shown in Fig. S1c show the characteristic (002) peak of $Ti_3C_2T_x$ MXene at ~7° 2θ, confirming successful MAX phase etching, while the Ti_3AlC_2 precursor exhibits expected MAX phase peaks at 9.5°, 18.9°, and 38.9° corresponding to (002), (004), and (006) reflections, respectively[3-5].

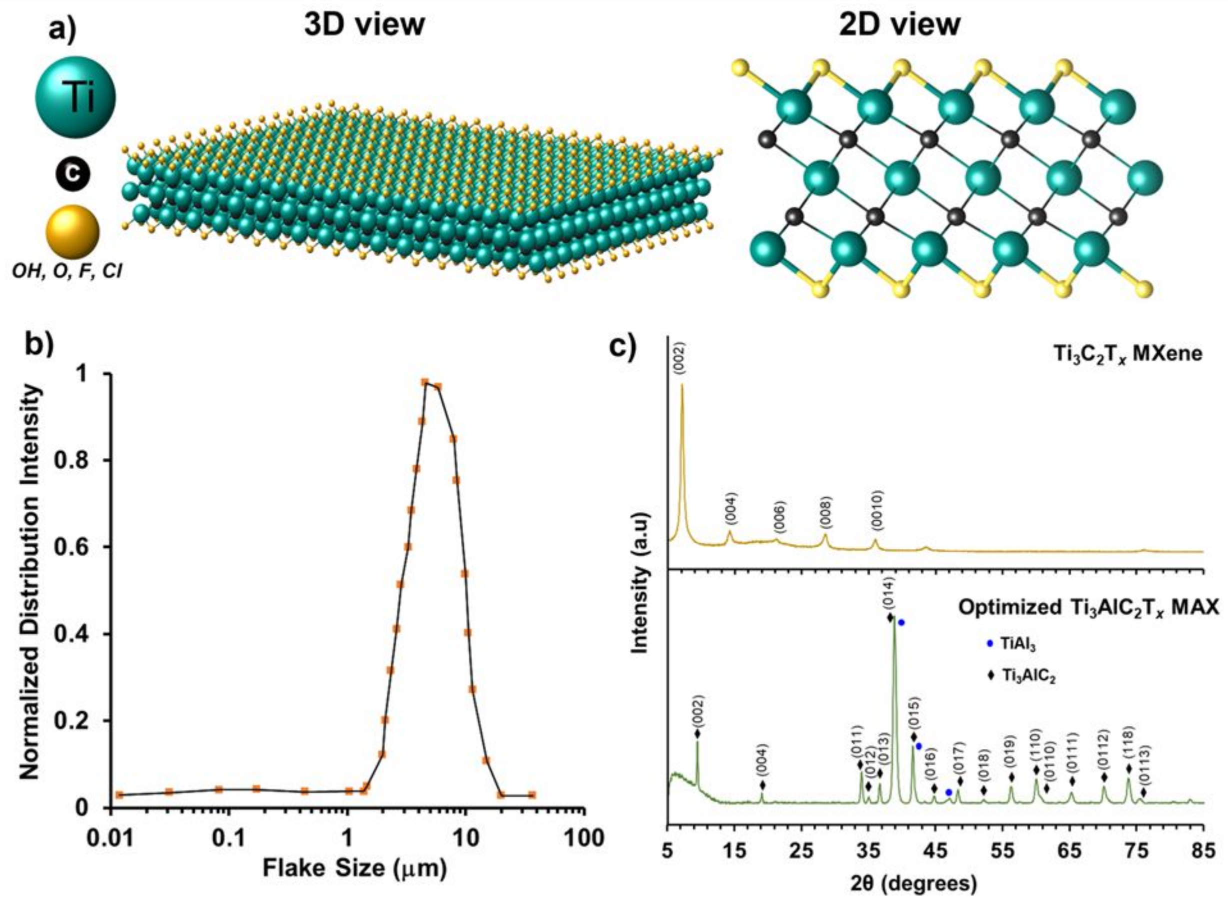

Figure S1. $Ti_3C_2T_x$ MXene characterization. (a) Atomic structure schematic showing 2D carbide lattice with surface terminations (-OH, -O, -F, -Cl). (b) Particle size distribution by laser diffraction showing narrow distribution (5-10 μm) with minimal submicron particles. (c) XRD patterns of $Ti_3C_2T_x$ MXene showing characteristic (002) peak at ~7° 2θ and Ti_3AlC_2 MAX precursor showing typical MAX phase reflections.

S2. Fabrication of PVDF and $Ti_3C_2T_x$ MXene-PVDF Composite Membranes

PVDF and $Ti_3C_2T_x$ MXene-PVDF composite membranes were fabricated via nonsolvent-induced phase separation (NIPS) following the procedure illustrated in Figure S2. Three membrane compositions were prepared: pristine PVDF (0 wt% MXene), 0.5 wt% MXene-PVDF, and 1 wt% MXene-PVDF. $Ti_3C_2T_x$ MXene dispersions were prepared by adding the calculated mass of delaminated MXene nanosheets to N,N-dimethylacetamide (DMAc, anhydrous, 99.8%, Sigma-Aldrich) and sonicating for 30 minutes using a probe sonicator (20 kHz, 50% amplitude) to ensure uniform dispersion. PVDF powder (Sigma Aldrich, USA, M.W. ~ 534,000 $g \cdot mol^{-1}$) was then added to the MXene-DMAc dispersion at a concentration of 16 wt% and stirred magnetically at 85 °C for 8 h until complete dissolution. The resulting homogeneous dope solution was degassed overnight under vacuum to remove entrapped air bubbles. Hydrophobic PTFE support membranes of 0.45 μm pore size obtained from Sterlitech were secured on a clean glass plate. The degassed polymer solution was cast onto the PTFE substrate using a doctor blade set to 200 μm gap height. The cast film was immediately immersed in a deionized water coagulation bath at room temperature of 23 ± 2 °C, initiating nonsolvent-induced phase separation. The membrane remained in the water bath for 12 h to ensure complete solvent exchange and phase inversion. The formed composite membranes were carefully removed from the coagulation bath, rinsed thoroughly with fresh deionized water to remove residual solvent, and air-dried overnight in a dust-free environment. Dried membranes were stored between clean sheets of filter paper in sealed containers to prevent contamination prior to characterization and testing.

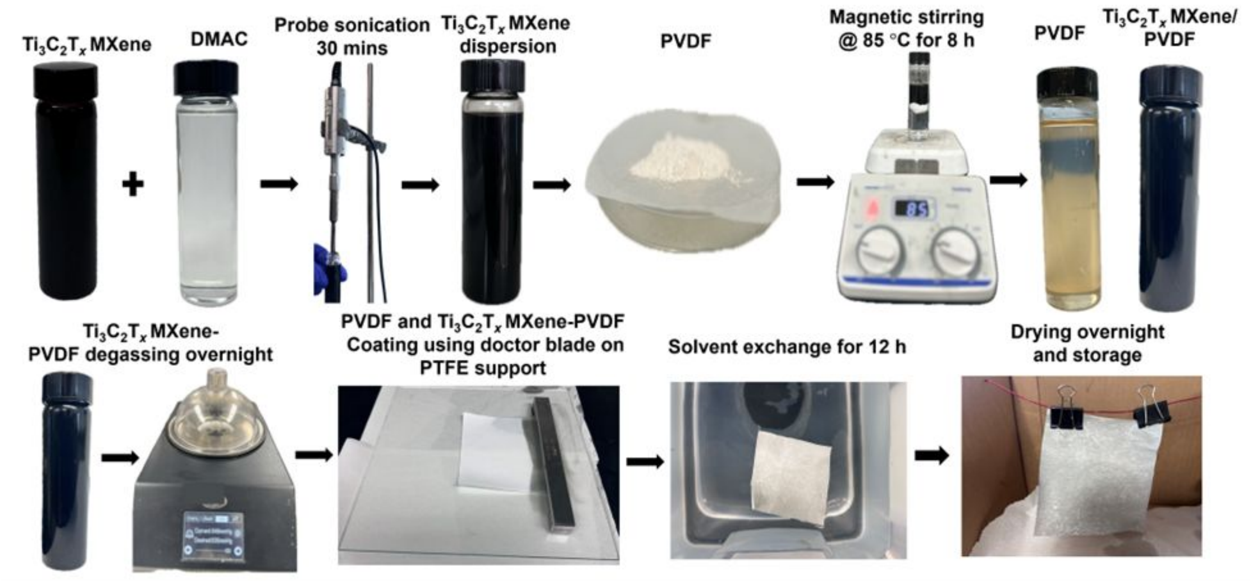

Figure S2. Schematic illustration of $Ti_3C_2T_x$ MXene-PVDF composite membrane fabrication via nonsolvent-induced phase separation. Sequential steps: (1) MXene dispersion in DMAc via probe sonication, (2) PVDF dissolution and magnetic stirring at 85 °C for 8 h, (3) degassing overnight under vacuum, (4) doctor blade casting onto PTFE support (200 µm gap), (5) immersion precipitation in water bath for 12 h, (6) drying and storage.

S3. Contact angle measurements for PVDF and $Ti_3C_2T_x$ MXene-PVDF composite membranes

Advancing and receding water contact angles were measured on PVDF, 0.5 wt% $Ti_3C_2T_x$ MXene-PVDF, and 1 wt% $Ti_3C_2T_x$ MXene-PVDF membranes using a Theta Lite optical tensiometer by Biolin Scientific with a controlled water dispense rate of 0.5 $\mu L \cdot s^{-1}$. Membranes were mounted on clean glass slides and equilibrated at ambient conditions of 23 ± 2 °C and 40-60 % relative humidity for 30 minutes prior to measurement. Dynamic contact angle evolution was recorded at 10 frames per second over a 10 s observation period, with automated droplet profile analysis using OneAttension software. Dynamic contact angles recorded over 10 s shown in

Figure S3a revealed distinct wetting behavior across membrane compositions. The pristine PVDF membrane exhibited low advancing contact angle of approximately 83° and receding angle of approximately 20°, reflecting limited wetting resistance and significant contact line pinning characteristic of moderate hydrophobicity. In contrast, $Ti_3C_2T_x$ MXene-PVDF composites demonstrate markedly enhanced dynamic wetting resistance. The 0.5 wt% MXene loading yields are advancing and receding angles of 89° and 65°, respectively, while the 1 wt% loading achieves 104° and 75°, indicating progressively enhanced hydrophobicity and substantially reduced contact angle hysteresis seen in Figs S3b-d. The systematic improvement in wetting characteristics with increasing MXene content confirms successful surface energy modification through 2D material integration. These results are consistent with Gibbs excess surface thermodynamics and validate the modified Cassie-Baxter framework discussed in the main manuscript, where hierarchical surface roughness and optimized interfacial energetics combined to enhance liquid repellency.

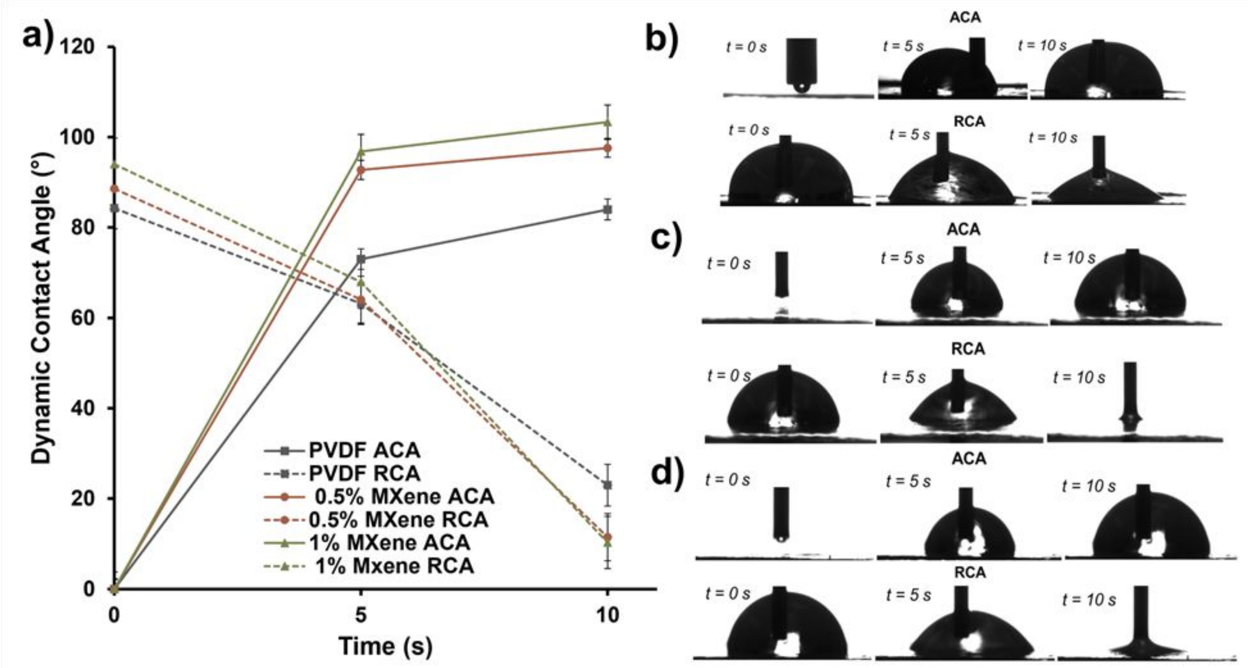

Figure S3. Dynamic contact angle measurements. (a) Temporal evolution of advancing (solid lines) and receding (dashed lines) contact angles over 10 s for PVDF, 0.5 wt% MXene-PVDF, and 1 wt% MXene-PVDF membranes. (b-d) Representative image sequences showing advancing and receding contact angle dynamics at t = 0, 5, and 10 s for: (b) pristine PVDF, (c) 0.5 wt% MXene-PVDF, and (d) 1 wt% MXene-PVDF membranes, demonstrating progressive hydrophobicity enhancement and reduced contact line pinning.

S4. Liquid entry pressure (LEP) measurement of PVDF and $Ti_3C_2T_x$ MXene-PVDF composite membranes

Liquid entry pressure (LEP) measurements were conducted using a custom dead-end filtration apparatus to assess membrane wetting resistance, shown schematically in Figure S4. The experimental setup consists of a membrane filtration cell obtained from Sterlitech HP4750, compressed air supply with precision pressure regulation, digital pressure gauge at ±0.5 kPa accuracy, and collection vessel for permeate detection. Membrane samples with an effective filtration area of 14.6 cm^2) were mounted in the filtration cell with appropriate O-ring sealing to prevent edge bypass. The upper chamber was filled with deionized water maintained at pH 6.5-7.0, conductivity <5 μS/cm at room temperature. Compressed air pressure was applied to the feed water and increased incrementally at a rate of 2 kPa/min using a precision pressure regulator valve. A digital pressure gauge monitored applied pressure with continuous data logging at 1s intervals. The membrane underside was visually monitored using LED backlighting to detect the first appearance of liquid breakthrough. The liquid entry pressure was defined as the applied pressure at which the first permeate droplet appeared on the membrane underside, indicating initial pore wetting and breakthrough. Each membrane was tested in triplicate, with fresh membrane samples used for each measurement to avoid hysteresis effects. The system was depressurized between

tests and allowed to equilibrate for 5 minutes before the next measurement. LEP values are reported as the average of three independent measurements with standard deviation. The measured LEP values validate the Young-Laplace equation relationship between surface properties and wetting resistance, confirming that $Ti_3C_2T_x$ MXene integration enhances membrane hydrophobicity without compromising structural integrity. These measurements provide critical validation for the interfacial thermodynamics framework discussed in the main manuscript.

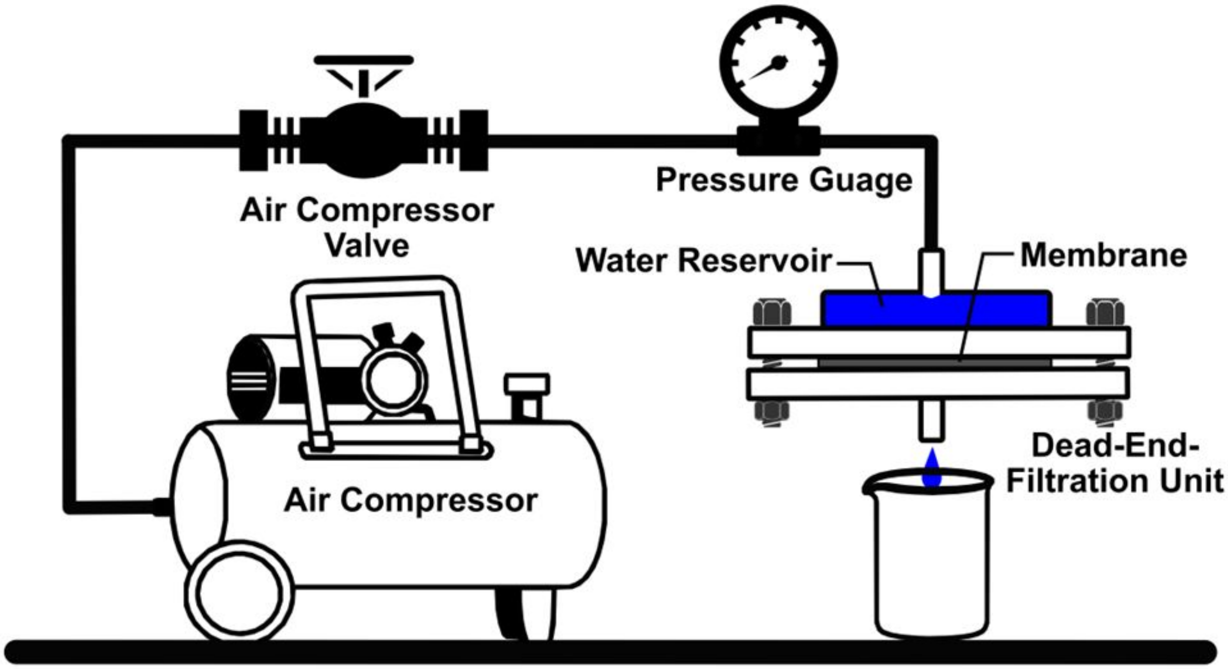

Figure S4. Liquid entry pressure measurement apparatus. Schematic of dead-end filtration setup showing compressed air supply, pressure regulation valve, digital pressure gauge, membrane filtration cell, and permeate collection vessel for breakthrough detection.

S5. Maxwell-Stefan transport model predictions

Using the parameters summarized in Table S1, we evaluated Equations 6-13 to compute the Knudsen contribution J_K, the molecular (Maxwell-Stefan) contribution J_M, and the composite predicted water-vapor mass flux Jacross the membrane for the best performing membrane (1% MXene-PVDF/PTFE).

Table S1. Input parameters along with its values used for theoretically predicted flux using integrated Maxwell-Stephen model.

Parameter	Symbol	Value	Units	Source
Porosity	ε	81.4	%	Measured
Mean pore radius	r_p	0.105	µm	Measured
Membrane thickness	δ_m	225	µm	Measured ($\pm$ 2.4)
Molar mass (H_2O)	M	0.018	kg/mol	Constant
Gas constant	R	8.314	J/mol·K	Constant
Tortuosity	τ	2	-	Literature[6]
Temperature	T	343.15	K	Operating conditions
H_2O-air diffusivity	$D_{w,air}$	2.77×10^{-5}	m^2/sec	Chapman-Enskog theory[7]
Vapor ΔP	ΔP_{vap}	28.9	kPa	Calculated

Total pressure	P_{tot}	31.7	kPa	Calculated
Feed pressure (air)	$P_{air,1}$	0.5	kPa	Calculated
Permeate pressure (air)	$P_{air,2}$	29.3	kPa	Calculated
Log-mean air pressure	$P_{air,ln}$	7	kPa	Calculated

Calculations were performed under the following standard assumptions: steady-state, one-dimensional transport; isothermal pores at the mean operating temperature; ideal-gas mixture behavior; binary diffusion of water vapor through stagnant air; negligible viscous flow relative to diffusive transport; spatially uniform and constant porosity ε, mean pore radius r_p, and tortuosity τthrough the thickness; cylindrical pores with diffuse molecule–wall reflections for Knudsen transport; no pore blocking, wetting, or capillary condensation within the pores; constant properties evaluated at the stated temperature; negligible external gas-phase boundary-layer resistance on both sides; negligible thermal diffusion and thermal transpiration effects; and no chemical reactions or accumulation within the membrane. The resulting values of J_K, J_M, and the combined flux $J$are reported in Table S2.

Table S2. Knudsen, Molecular and predicted flux using integrated Maxwell-Stephen model.

**Parameter**	**Symbol**	**Value ($kg{\cdot}m^{-2}{\cdot}h^{-1}$)**
Knudsen flux	J_K	52.7
Molecular flux	J_M	149.7

Predicted flux	J_{Pred}	39.1
Experimental flux	J_{Exp}	42

S6. Thermal Efficiency Calculations and Sensitivity Analysis

Thermal efficiency was calculated across a parametric grid of vapor flux values J = 20-70 $kg \cdot m^{-2} \cdot h^{-1}$ and effective membrane thermal conductivity (k_m range $W \cdot m^{-1} \cdot K^{-1}$ at constant ΔT = 50°C, δ_m = 225 μm, and ΔH_v = 2394.6 $kJ \cdot kg^{-1}$ and shown in Figure S5. The color scale transitions from red, low η, conduction-dominated through yellow to green with high η, evaporation-dominated. The heatmap revealed several important trends, first, thermal efficiency is strongly dependent on vapor flux: at any given k_m, increasing J from 20 to 70 $kg \cdot m^{-2} \cdot h^{-1}$ improves η by approximately 20-30 percentage points, reflecting the growing dominance of q_v over q_c at higher permeation rates. Second, reducing k_m from 0.180 to 0.030 $W \cdot m^{-1} \cdot K^{-1}$ at constant flux increases η by approximately 30-40 percentage points, confirming that minimizing conductive heat loss through increased porosity and air-filled void fraction is a critical design strategy for thermally efficient membranes. Third, the combined effect is synergistic: the highest efficiencies (η > 80%) are achieved only when both high flux ($J \geq 50$ $kg \cdot m^{-2} \cdot h^{-1}$) and low thermal conductivity ($k_m \leq$ 0.060 $W \cdot m^{-1} \cdot K^{-1}$) are simultaneously realized, a design region that corresponds to high-porosity membranes with strong hydrophobic character. For the three membrane compositions studied in this work, the operating points fall along a diagonal trajectory from the lower-left (PVDF control: J = 23.4 $kg \cdot m^{-2} \cdot h^{-1}$, k_m = 0.073 $W \cdot m^{-1} \cdot K^{-1}$, η ~ 49%) to the center-right (1 wt% MXene-PVDF: J = 42.0 $kg \cdot m^{-2} \cdot h^{-1}$, k_m = 0.057 $W \cdot m^{-1} \cdot K^{-1}$, η ~ 68%) of the parametric space, confirming that MXene incorporation simultaneously increases flux and decreases thermal conductivity, a thermodynamically favorable design trajectory. The 1 wt% MXene-PVDF membrane falls within

the green-shaded high-efficiency zone (η = 65-75%), consistent with well-performing DCMD membranes reported in the literature. Further improvements in η toward the upper-right corner of the design space (η > 80%) would require either higher flux (through optimized pore geometry or reduced membrane thickness) or further reductions in k_m (through higher porosity or air-gap configurations), representing quantifiable targets for next-generation membrane design.

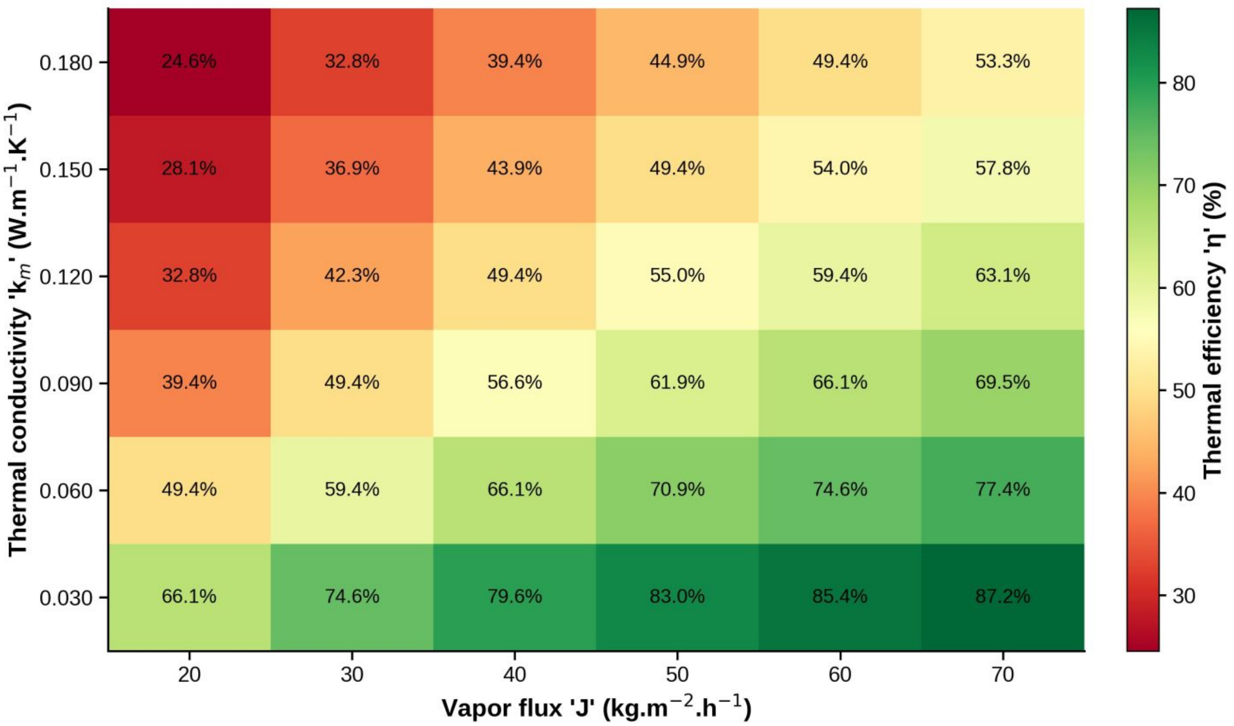

Figure S5: Parametric heatmap of thermal efficiency (η, %) as a function of vapor flux (J, $kg\cdot m^{-2}\cdot h^{-1}$) and effective membrane thermal conductivity (k_m, $W\cdot m^{-1}\cdot K^{-1}$), calculated at ΔT = 50°C and δ_m = 225 μm. The color gradient transitions from red (η < 30%, conduction-dominated) to green (η > 80%, evaporation-dominated). Operating points for the PVDF control, 0.5 wt%, and 1 wt% MXene-PVDF membranes fall along a favorable diagonal trajectory from the lower-left to the center-right of the design space.

The thermal efficiency and transport model predictions depend on parameters that are either assumed from the literature for tortuosity τ, solid-phase thermal conductivity k_{solid} or subject to measurement uncertainty such as temperature polarization. Each parameters was systematically varied independently to quantify its influence on the reported results. Figure S6 presents the dependence of calculated thermal efficiency on the assumed tortuosity value, varied from $\tau = 1.2$ to $\tau = 4.0$, while all other parameters are held constant at their baseline values ($\varepsilon = 81.4\%$, $r_p = 0.105$ μm, $\delta_m = 225$ μm, $k_m = 0.057$ $W \cdot m^{-1} \cdot K^{-1}$, $\Delta T = 50°C$). Tortuosity directly controls the predicted vapor flux through the combined Knudsen-molecular diffusion transport model ($J \propto \varepsilon/\tau\delta_m$), thereby modulating the evaporative heat flux q_v and consequently the thermal efficiency. The relationship between η and τ is monotonically decreasing and concave, reflecting the inverse proportionality between tortuosity and vapor flux. At the lowest value explored ($\tau = 1.2$), η reaches approximately 77%, corresponding to a scenario in which pores are nearly straight, and transport resistance is minimal. As τ increases toward 4.0, η declines to approximately 50%, at which point evaporative and conductive heat fluxes are nearly equal and the membrane approaches the threshold of conduction-dominated thermal behavior. The rate of decline is steepest in the low-τ region ($\tau = 1.2$ to 2.5), where each 0.5-unit increment in τ reduces η by approximately 4 to 5 percentage points, and progressively flattens at higher τ values as the evaporative contribution asymptotically diminishes. At the baseline assumption of $\tau = 2.0$ (standard literature value for isotropic NIPS-fabricated membranes), the calculated thermal efficiency is approximately 66%, which falls within the expected range for bench-scale DCMD systems (40-70%) and is consistent with the values reported in the main manuscript. The predicted flux at this tortuosity (39.0 $kg \cdot m^{-2} \cdot h^{-1}$) deviates by only -7.2% from the experimentally measured value of 42.0 $kg \cdot m^{-2} \cdot h^{-1}$, providing independent validation that $\tau = 2$ is a physically appropriate assumption for these

membranes. Importantly, even under the most conservative assumption ($\tau = 4.0$), η remains at approximately 50%, confirming that the membrane maintains acceptable thermal performance across the full credible range of tortuosity values and that the energy performance conclusions presented in the main manuscript are robust to this assumed parameter.

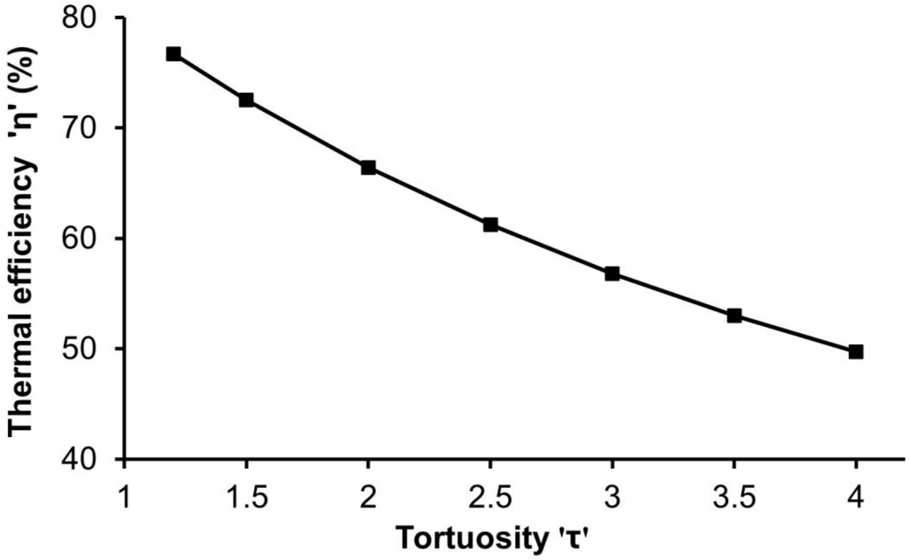

Figure S6. Sensitivity of thermal efficiency (η, %) to assumed tortuosity ($\tau = 1.2$-4.0) for the 1 wt% MXene-PVDF-PTFE composite membrane. Calculations performed at constant $\varepsilon = 81.4\%$, $\delta_m = 225$ μm, $k_m = 0.057$ $W \cdot m^{-1} \cdot K^{-1}$, and $\Delta T_{bulk} = 50°C$. The baseline value $\tau = 2$ yields $\eta \sim 66\%$ and a predicted flux within 7.2% of the measured value, validating the assumed tortuosity. η remains above 50% across the full parametric range

All sensitivity calculations were performed for the 1 wt% $Ti_3C_2T_x$ MXene-PVDF membrane. Tortuosity (τ) appears in the denominator of the transport model ($J \propto \varepsilon/\tau\delta_m$) and directly scales the predicted flux. Since τ was not measured independently but assigned a value of 2 based on the commonly used approximation for isotropic porous membranes, its sensitivity must

be assessed. Table S3 reports the predicted flux and its deviation from the experimentally measured value of 42.0 kg·m^{-2}·h^{-1} as τ is varied from 1.2 to 4.0.

Table S3. Predicted vapor flux as a function of tortuosity for the 1 wt% $Ti_3C_2T_x$ MXene-PVDF membrane (ε = 81%, J = 42 kg·m^{-2}·h^{-1}, ΔT = 50°C). Measured flux: kg·m^{-2}·h^{-1}.

**Tortuosity (τ)**	**Predicted flux (kg·m^{-2}·h^{-1})**	**Deviation from Experimental flux (%)**	**Agreement**
1.2	65.00	54.8	Poor
1.5	52.00	23.8	Moderate
2.0	39.00	7.1	Good
2.5	31.20	25.7	Moderate
3.0	26.00	38.1	Poor
3.5	22.29	46.9	Poor
4.0	19.50	53.6	Poor

The predicted flux varies strongly with τ, confirming that tortuosity is the most influential assumed parameter in the transport model. At the assumed value of τ = 2, the model predicted 39 kg·m^{-2}·h^{-1}, deviating by 7.1% from the measured 42 kg·m^{-2}·h^{-1}. This is the closest agreement among all tested values and falls within the ~ 5-30% range typical of dusty-gas model predictions for polymeric membranes. Tortuosity values below 1.5 or above 2.5 produce deviations exceeding ±30% and can be excluded on physical grounds for these membrane morphologies. The result validates τ = 2 as a reasonable and well-justified assumption for the NIPS-fabricated membranes in this study.

The solid-phase thermal conductivity (k_{solid}) controls the magnitude of conductive heat loss and therefore directly affects the computed η. The baseline value of k_{solid} = 0.19 $W \cdot m^{-1} \cdot K^{-1}$ corresponds to neat PVDF. However, the presence of $Ti_3C_2T_x$ MXene nanosheets within the polymer matrix could increase the effective solid-phase conductivity due to MXene's high intrinsic thermal conductivity. Table S4 examines the sensitivity of η to ksolid over the range 0.10 to 0.30 $W \cdot m^{-1} \cdot K^{-1}$, spanning from a conservative lower bound to an upper bound accounting for possible MXene thermal percolation effects. Thermal efficiency exhibits moderate sensitivity to k_{solid}, varying from 61% at the lower bound to 75% at the upper bound, a total range of approximately 14%. Even under the most pessimistic assumption (k_{solid} =0.3 $W \cdot m^{-1} \cdot K^{-1}$, representing substantial thermal percolation through MXene), η remains above 60%, indicating that the high evaporative heat flux dominates the energy balance across the entire plausible parameter range. This moderate sensitivity arises because the membrane porosity is high, ε = 81%, so the gas-phase conductivity contributes the majority of k_m regardless of the solid-phase value. The baseline η = 68% reported in the main text is therefore a robust estimate that is not critically dependent on the precise value of k_{solid}.

Table S4. Thermal efficiency as a function of solid-phase thermal conductivity for the 1 wt% $Ti_3C_2T_x$ MXene-PVDF membrane (ε = 81%, J = 42 $kg \cdot m^{-2} \cdot h^{-1}$, ΔT = 50°C).

**k_{solid} ($W \cdot m^{-1} K^{-1}$)**	**k_m ($W \cdot m^{-1} \cdot K^{-1}$)**	**q_c ($kW \cdot m^{-2}$)**	**η (%)**	**Physical basis**
0.1	0.0409	9.08	75	Lower bound (low-k polymer)
0.15	0.0504	11.19	70.9	Fluoropolymer range
0.19	0.0580	12.88	67.9	Baseline (neat PVDF)
0.25	0.0694	15.42	63.8	Moderate MXene contribution

0.3	0.0789	17.53	60.8	Upper bound (high MXene loading)

Temperature polarization is the largest source of uncertainty in the thermal efficiency estimate. Because direct membrane surface temperature measurements were not performed (thermocouples were positioned at the module inlet and outlet), the transmembrane ΔT used for q_c estimation was taken as the bulk value of 50°C. In reality, temperature polarization reduces the effective ΔT at the membrane surface. The temperature polarization coefficient, $TPC = \Delta T_{eff} / \Delta T_{bulk}$, typically ranges from 0.4 to 0.9 for DCMD, with values of 0.6 to 0.8 being most common for laminar flat-sheet configurations. Critically, adjusting for temperature polarization reduces q_c while q_v remains fixed at the experimentally measured value (because q_v is calculated directly from the measured permeate flux, not from the assumed ΔT). Temperature polarization therefore increases the calculated η. Table S5 quantifies this effect.

Table S5. Thermal efficiency as a function of temperature polarization coefficient for the 1 wt% $Ti_3C_2T_x$ MXene-PVDF membrane (q_v = 27.2 kW·m^{-2} fixed).

**TPC**	***ΔT_{eff}* *(°C)***	***q_c*** **(kW·m^{-2})**	***η*** **(%)**	**Notes**
1.0	50	12.9	68	No polarization (baseline)
0.9	45	11.6	70.1	Mild polarization
0.8	40	10.3	72.5	Most probable range
0.7	35	9	75.1	Most probable range
0.6	30	7.7	77.9	Common for laminar flat-sheet DCMD
0.5	25	6.4	80.9	Severe polarization

0.4	20	5.1	84.1	Severe polarization

At the most probable TPC range of 0.6 to 0.8 for the laminar crossflow conditions used in this study (Re < 400, flat-sheet module), the thermal efficiency of the 1 wt% MXene-PVDF membrane increases to approximately 75%, compared with the conservative baseline of 68% at $TPC = 1.0$. The η values reported in the main text therefore represent lower-bound estimates, and the true membrane thermal efficiency is likely several percentage points higher. The sensitivity analysis supports that the tortuosity value of $\tau = 2$ produces the closest model-experiment agreement (-7.2% deviation) among all values tested and is well justified for isotropic porous membranes fabricated by NIPS. The thermal efficiency is moderately sensitive to the assumed solid-phase thermal conductivity and the choice of mixing model, but η remains above 60% across the entire plausible parameter space and above 68% at the baseline values used in this study. Third, temperature polarization is the dominant source of uncertainty; accounting for realistic *TPC* values increases the estimated η from 68% to 73-78%. Future experiments incorporating direct membrane surface temperature measurements will enable more precise thermal efficiency determination and eliminate the need for *TPC* estimation**.**

S7. Surface roughness characterization of PVDF and $Ti_3C_2T_x$ MXene-PVDF composite membranes

Surface roughness was quantified using a Keyence VHX-600 digital microscope with automated image analysis capabilities. Membrane samples were secured on clean glass slides and analyzed under standardized imaging conditions of 200X magnification, LED coaxial illumination, and autofocus enabled. For each membrane composition, five randomly selected

regions of interest (ROI) per sample were imaged with a minimum of 10 independent membranes analyzed per composition, with each ROI having dimensions of 500 × 500 μm. Three-dimensional surface profiles were reconstructed from optical height data, and the arithmetical mean height (S_a) was calculated according to ISO 25178-2 standards using integrated VHX software. The arithmetical mean height (S_a) increases progressively with $Ti_3C_2T_x$ MXene loading summarized in Table S1. Pristine PVDF membranes exhibit S_a of 5.5 ± 1.9 μm, whereas 0.5 wt% MXene-PVDF increases to 8.7 ± 0.8 μm showing 58% increase, and 1 wt% MXene-PVDF reaches 14.3 ± 3.5 μm with 160% increase. The progressive roughness enhancement reflected MXene nanosheet incorporation, which perturbed polymer chain packing during nonsolvent-induced phase separation and produces more pronounced surface topography[8, 9]. This systematic roughness increased with improved hydrophobicity and enhanced wetting resistance observed in contact angle measurements, validating the Cassie-Baxter prediction that surface roughness amplifies hydrophobic behavior[9, 10]. The reduced standard deviation at 0.5 wt% loading at 0.8 μm vs. 1.9 and 3.5 μm for PVDF and 1% MXene, respectively suggesting optimal surface homogeneity at intermediate MXene concentration, with increased heterogeneity at the highest loading potentially reflecting localized MXene aggregation or uneven distribution.

Table S3. Surface roughness measurements of PVDF and $Ti_3C_2T_x$ MXene-PVDF composite membranes.

**Membrane Composition**	**S_a (μm)**
PVDF	5.5 ± 1.9
0.5% $Ti_3C_2T_x$ MXene-PVDF	8.7 ± 0.8

1% $Ti_3C_2T_x$ MXene-PVDF	14.3 ± 3.5

S8. Elemental mapping of $Ti_3C_2T_x$ MXene-PVDF composite membranes

The 1% $Ti_3C_2T_x$ MXene-PVDF composite membrane surface was imaged using an energy Hitachi S-2400 SEM equipped with dispersive spectrometer EDS for elemental analysis. Gold sputtering was performed before the EDS analysis for inducing electrical conductivity to avoid charging effect during imaging. The elemental mapping of 1% $Ti_3C_2T_x$ MXene-PVDF composite membrane surface is shown in Figure S7. The presence of Ti and C confirmed the distribution of $Ti_3C_2T_x$ MXene on the membrane surface.

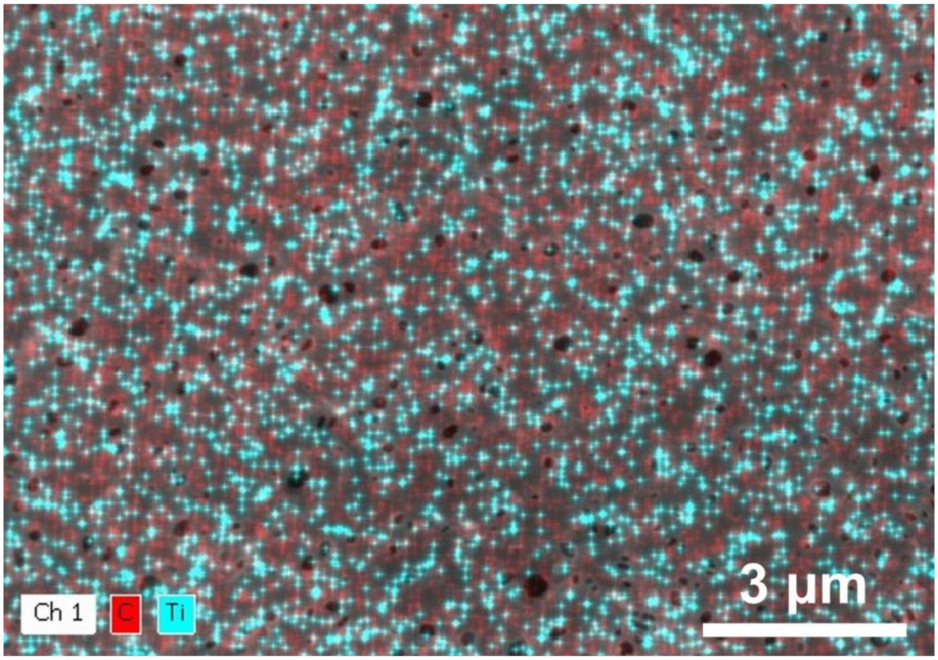

Figure S7. Elemental mapping of 1% $Ti_3C_2T_x$ MXene-PVDF composite membrane surface. Uniform distribution of C and Ti across the membrane surface confirms the presence of $Ti_3C_2T_x$ MXene.

S9. Porosity measurement using gravimetric method (ASTM D2873)

To determine the dry mass (m_d), membrane coupons were dried for 24 hours at 50°C, cooled to room temperature in a desiccator, and then weighed. After that, samples were submerged in ethanol for a full day to ensure full pore infiltration. The wet mass (m_w) was noted as soon as the excess surface liquid was gently removed using a standardized blotting process (lint free tissue, fixed time and pressure). The open porosity (ε) was computed as[11, 12]:

$$\varepsilon\,(\%) = \frac{m_w - m_d}{\rho_l A \delta} \times 100 \qquad [S1]$$

where ρ_l is the density of ethanol (0.7893 g cm^{-3} at the measurement temperature), A is the wet membrane area, and δ is the membrane thickness. Results were reported as the mean ± SD from at least three replicate specimens.

S10. DigiM Analysis

DigiM I2STM (DigiM Solution LLC, Burlington, MA), a cloud-hosted platform with a web interface for accessibility, usability, and integrated data management, was used to rebuild and analyze three-dimensional SEM image stacks[13]. To meet the significant computational requirements of image-based computational fluid dynamics (CFD) calculations and artificial intelligence (AI)-based image segmentation, the platform makes use of a distributed-memory parallel computing cluster. Then, quantitative analysis was performed using AI-based image segmentation of features of interest into distinct materials phases[14]. The AI based image segmentation algorithm implements a supervised machine learning framework. As a training image, a small 2D subset of the representative of the entire 3D stack with the necessary characteristics and typical imaging artifacts is chosen[15]. Each target feature class is then annotated

by a human operator. Next, the system used a bank of image filters that record spatial context and textural interactions between nearby pixels in addition to pixel intensity to acquire class discriminative signatures. For every material phase in relation to the imaging signal, these filter-derived responses form a textural fingerprint[16]. The trained model was run on the server to automatically segment any required number of photos after about ten to fifteen minutes of iterative training and qualitative user evaluation of segmentation fidelity. As long as the imaging settings were calibrated and the samples had identical material classes, this automated approach could be extended to the remaining slices within the same SEM stack or across various stacks. Following phase segmentation, quantitative morphometrics, such as pore size distribution, were calculated for each phase to produce statistical descriptors that more accurately describe the sample microstructure numerically[16].

References:

(1) Thakur, A.; Zhang, Y.; Gogotsi, Y.; Anasori, B. Electrochemistry of MXenes and their sustainable energy applications. *MRS energy & sustainability* **2025**, *12* (2), 1-13.

(2) Thakur, A.; Chandran BS, N.; Davidson, K.; Bedford, A.; Fang, H.; Im, Y.; Kanduri, V.; Wyatt, B. C.; Nemani, S. K.; Poliukhova, V. Step-by-step guide for synthesis and delamination of Ti3C2Tx MXene. *Small Methods* **2023**, *7* (8), 2300030.

(3) Merugu, S.; Hasan, M. M.; Thakur, A.; Patenaude, J.; Anasori, B.; Choueiri, G.; Gupta, A. Ti3C2T x MXene Additives for Enhanced Pool Boiling Regime. *Acs Omega* **2025**, *10* (7), 6534-6543.

(4) Ma, G.; Zhang, A.; Wang, Z.; Wang, K.; Zhang, J.; Xu, K.; Xu, Y.; Zhou, S.; Wang, A. MAX phase coatings: synthesis, protective performance, and functional characteristic. *Materials Horizons* **2025**.

(5) Alam, M. S.; Chowdhury, M. A.; Khandaker, T.; Hossain, M. S.; Islam, M. S.; Islam, M. M.; Hasan, M. K. Advancements in MAX phase materials: structure, properties, and novel applications. *RSC advances* **2024**, *14* (37), 26995-27041.

(6) Cramer, K.; Prasianakis, N. I.; Niceno, B.; Ihli, J.; Holler, M.; Leyer, S. Three-dimensional membrane imaging with X-ray ptychography: determination of membrane transport properties for membrane distillation. *Transport in Porous Media* **2021**, *138* (2), 265-284.

(7) Langenberg, S.; Carstens, T.; Hupperich, D.; Schweighoefer, S.; Schurath, U. Determination of binary gas-phase diffusion coefficients of unstable and adsorbing atmospheric trace gases at low temperature–arrested flow and twin tube method. *Atmospheric Chemistry and Physics* **2020**, *20* (6), 3669-3682.

(8) Wang, F.; Zhang, Z.; Shakir, I.; Yu, C.; Xu, Y. 2D polymer nanosheets for membrane separation. *Advanced Science* **2022**, *9* (8), 2103814.

(9) Zhou, H.; Wang, F.; Wang, Y.; Li, C.; Shi, C.; Liu, Y.; Ling, Z. Study on contact angles and surface energy of MXene films. *RSC advances* **2021**, *11* (10), 5512-5520.

(10) Zukiene, K.; Monastyreckis, G.; Kilikevicius, S.; Procházka, M.; Micusik, M.; Omastová, M.; Aniskevich, A.; Zeleniakiene, D. Wettability of MXene and its interfacial adhesion with epoxy resin. *Mater Chem Phys* **2021**, *257*, 123820.

(11) El-Nemr, H. A.; ElKady, M.; El-Khouly, M. E. Polystyrene-Modified PVDF Membranes: A Novel Approach for Improved Flux and Salt Rejection in Direct Contact Membrane Distillation. *Journal of Applied Polymer Science* **2026**, *143* (5), e58133.

(12) Boukhriss, M.; Maatoug, M. A.; Zarzoum, K. Experimental and numerical investigation of a solar-driven Air-Gap Membrane Distillation (AGMD) system for seawater desalination. *Journal of Engineering and Applied Science* **2026**, *73* (1), 49.

(13) Howard, J.; Lin, S.; Zhang, S. Uncertainty quantification in image segmentation for image-based rock physics in a shaly sandstone. *Petrophysics* **2019**, *60* (02), 240-254.
(14) Zhang, S.; Lomeo, J. Cloud-based image management solutions for digital transformation of drug product development. *Microscopy and Microanalysis* **2021**, *27* (S1), 296-297.
(15) Zhang, S.; Byrnes, A. P.; Jankovic, J.; Neilly, J. Management, analysis, and simulation of micrographs with cloud computing. *Microscopy Today* **2019**, *27* (2), 26-33.
(16) Zhang, S.; Byrne, G. Characterization of transport mechanisms for controlled release polymer membranes using focused ion beam scanning electron microscopy image-based modelling. *Journal of Drug Delivery Science and Technology* **2021**, *61*, 102136.
